# Golgi localization of the LIN-2/7/10 complex points to a role in basolateral secretion of LET-23 EGFR in the *Caenorhabditis elegans* vulval precursor cells

**DOI:** 10.1242/dev.194167

**Published:** 2021-03-05

**Authors:** Kimberley D. Gauthier, Christian E. Rocheleau

**Affiliations:** Division of Endocrinology and Metabolism, Department of Medicine, and Department of Anatomy and Cell Biology, McGill University; and the Metabolic Disorders and Complications Program, Centre for Translational Biology, Research Institute of the McGill University Health Centre, Montreal, QC H4A 3J1, Canada

**Keywords:** *Caenorhabditis elegans*, Vulva, LET-23, Cask, Lin7, APBA

## Abstract

The evolutionarily conserved LIN-2 (CASK)/LIN-7 (Lin7A-C)/LIN-10 (APBA1) complex plays an important role in regulating spatial organization of membrane proteins and signaling components. In *Caenorhabditis elegans*, the complex is essential for the development of the vulva by promoting the localization of the sole Epidermal growth factor receptor (EGFR) ortholog LET-23 to the basolateral membrane of the vulva precursor cells where it can specify the vulval cell fate. To understand how the LIN-2/7/10 complex regulates receptor localization, we determined its expression and localization during vulva development. We found that LIN-7 colocalizes with LET-23 EGFR at the basolateral membrane, whereas the LIN-2/7/10 complex colocalizes with LET-23 EGFR at cytoplasmic punctae that mostly overlap with the Golgi. Furthermore, LIN-10 recruits LIN-2, which in turn recruits LIN-7. We demonstrate that the complex forms *in vivo* with a particularly strong interaction and colocalization between LIN-2 and LIN-7, consistent with them forming a subcomplex. Thus, the LIN-2/7/10 complex forms on the Golgi on which it likely targets LET-23 EGFR trafficking to the basolateral membrane rather than functioning as a tether.

## Teaser

**Summary:** Expression and localization analyses revealed that LIN-10 recruits LIN-2 and LIN-7 to Golgi, consistent with targeting rather than tethering the epidermal growth factor receptor to the basolateral membrane in *Caenorhabditis elegans*.

## INTRODUCTION

The spatial organization of signaling pathways is essential for the proper activation and function of signal transduction cascades, particularly in polarized cells (e.g. epithelial cells or neurons), in which cellular components are segregated into distinct domains. Mislocalization of signaling components can cause ectopic signaling activation or loss of signaling, leading to inappropriate cell responses in cell cycle regulation, migration or survival that can result in disease, developmental disorders and cancer (Hung and Link, 2011).

**Fig. 1.**
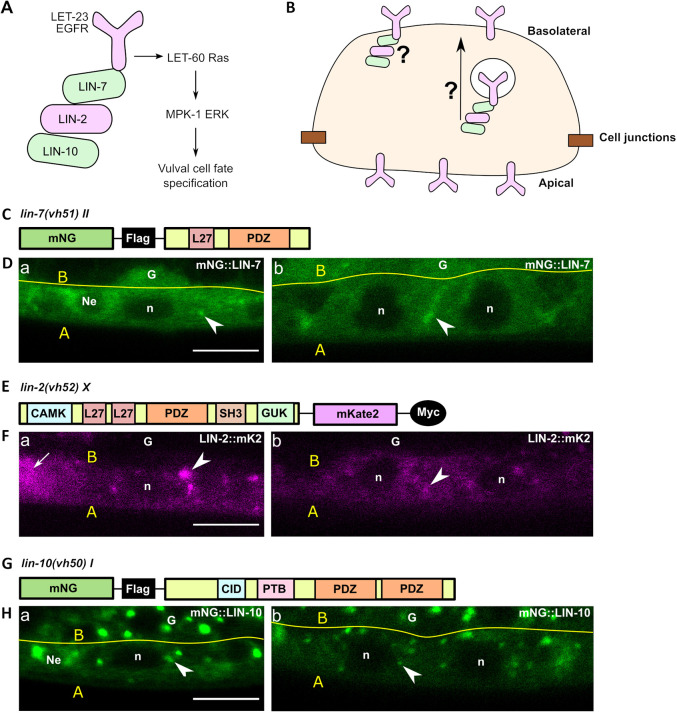
**Punctate localization of LIN-2, LIN-7 and LIN-10 in the VPCs.** (A) Schematic of how the LIN-2/7/10 complex interacts with the cytoplasmic tail of LET-23 EGFR in the VPCs, which is necessary for basolateral receptor localization and activation of the downstream Ras/ERK signaling cascade, which specifies the vulval cell fate. (B) Schematic of possible roles for the LIN-2/7/10 complex promoting LET-23 EGFR basolateral membrane localization as a membrane tether or via sorting on endomembrane compartments. (C,E,G) Schematic of endogenously tagged *lin-7* (C), *lin-2* (E) and *lin-10* (G) alleles generated by CRISPR/Cas9: *vh51*, *vh52* and *vh50*, respectively. (D,F,H) Fluorescent images of mNG::LIN-7 (D), LIN-2::mK2 (F) and mNG::LIN-10 (H) in P6.p cells at the early L2 stage (Da,Fa,Ha) and P6.px cells in the mid L3 stage (Db,Fb,Hb). Arrow indicates a segment of ventral nerve chord in same focal plane as the VPCs. Arrowhead in Da, F and H indicates punctate localization. Arrowhead in Db indicates lateral membrane localization. A, apical; B and yellow line, basal lamina of VPCs; G, somatic gonad primordium; mNG, mNeonGreen; n, nucleus; Ne, neuron. Scale bars: 5 μm.

A classic example of spatial regulation of cell signaling is that of *Caenorhabditis elegans* vulva development. During the L2 and L3 larval stages, the Epidermal growth factor receptor (EGFR) homolog LET-23 activates a downstream LET-60 Ras/MPK-1 ERK signaling cascade that specifies the primary vulval cell fate in the vulva precursor cells (VPCs) (Sternberg, 2005). Of the six VPCs, only three cells are induced to generate the vulva due to their proximity to the source of the EGF-like ligand LIN-3, which is secreted from the overlying gonadal anchor cell. The remaining three VPCs assume the uninduced cell fate and divide once before fusing with the surrounding hypodermal syncytium Hyp7. An evolutionarily conserved LIN-2 (CASK), LIN-7 (Lin7) and LIN-10 (APBA1) complex (the LIN-2/7/10 complex) is required for basolateral localization of LET-23 EGFR in the VPCs, adjacent to the anchor cell (Fig. 1A) (Kaech et al., 1998). Loss-of-function mutations in *lin-2*, *lin-7* or *lin-10*, or loss of interaction between LET-23 EGFR and LIN-7, results in exclusive apical localization of LET-23 EGFR, loss of signaling activation in the VPCs, and a vulvaless (Vul) phenotype (Ferguson and Horvitz, 1985; Aroian et al., 1994; Kaech et al., 1998). The interactions between components of the LIN-2/7/10 complex and LET-23 are well-defined: *in vitro* interaction assays and yeast two-hybrid assays have shown that the C-terminal PDZ-interaction motif of LET-23 EGFR interacts with the PDZ-domain of LIN-7 (Simske et al., 1996; Kaech et al., 1998; Shelly et al., 2003). LIN-7 interacts with LIN-2, which in turn interacts with LIN-10 (Kaech et al., 1998) (Fig. 1A).

Despite its characterization over 20 years ago, we still do not know where and how the LIN-2/7/10 complex functions to regulate LET-23 EGFR basolateral membrane localization. The complex might help anchor the receptor to the basolateral membrane, or work in the recycling or secretory pathways to target the receptor to the basolateral membrane (Fig. 1B). Furthermore, previous localization studies found that overexpressed LIN-7 overlapped with the apical junction marker (AJM-1) in the VPCs (Simske et al., 1996), whereas LIN-10 immunolocalized to cytoplasmic punctae in descendants of the VPCs (Whitfield et al., 1999), suggesting that they may occupy distinct subcellular compartments, which would be inconsistent with their functioning as a complex.

The mammalian homologs calcium/calmodulin-dependent serine protein kinase (CASK), Lin7 and amyloid beta precursor protein binding family A member 1 (APBA1) also regulate polarized membrane protein localization in both neurons and epithelial cells. Interactions between CASK, Lin7 and APBA1 have been validated in murine models (Borg et al., 1999, 1998; Butz et al., 1998), and the complex was co-immunoprecipitated from murine synaptic plasma membrane fractions (Butz et al., 1998). In neurons, the CASK/Lin7/APBA1 complex maintains synaptic localization of the NMDA receptor subunit NR2B (Jo et al., 1999; Setou et al., 2000; Jeyifous et al., 2009), the synaptic adhesion molecule neurexin (Butz et al., 1998; Fairless et al., 2008) and the G-protein coupled receptor 5-HT2C (Becamel et al., 2002). Lin7 and CASK are also expressed in epithelial cells, in which they interact and coordinate polarized localization of the potassium channel Kir2.3 (Alewine et al., 2007). APBA1 has recently been found to be expressed in epithelial tissue (Motodate et al., 2016); whether it forms a complex with CASK and Lin7 in mammalian epithelia has not been shown.

Localization studies in neurons and epithelial cells suggest multiple possible functions for the CASK/Lin7/APBA1 complex. Lin7 and CASK colocalize on the basolateral membranes of epithelial cells (Cohen et al., 1998; Straight et al., 2000; Stetak et al., 2006), consistent with a role in anchoring proteins at the basolateral membrane. Consistent with this, CASK can be anchored to the cell periphery through an interaction with actin-associated protein 4.1 to stabilize cargo localization to the plasma membrane (Cohen et al., 1998; Biederer and Südhof, 2001). In contrast, CASK also colocalizes with Golgi-associated APBA1 in neurons (Borg et al., 1999), suggesting a function in targeted secretion. Individually, CASK and all three APBA paralogs have been shown to localize to the nucleus and regulate transcription (Hsueh et al., 2000; Sumioka et al., 2008; Lau et al., 2010; Hirose et al., 2014; Swistowski et al., 2009), adding another layer of functional complexity. Colocalization between Lin7 and APBA1 has not been demonstrated. The disparate localization of CASK, Lin7 and APBA1 in epithelial and neuronal cells suggests that the complex could function in anchoring proteins at the basolateral membrane/synapses, polarized secretion from the Golgi, or even play a role in transcription.

The expression and subcellular localization of the LIN-2/7/10 complex in *C. elegans* has largely been unexplored and its function remains unknown. Identifying the subcellular localization of the LIN-2/7/10 complex formation, and where the complex localizes with LET-23 EGFR, will provide insight into how the complex regulates polarized receptor localization, such as by tethering LET-23 EGFR at the basolateral membrane or through targeted secretion. Here, we used CRISPR/Cas9 genome editing to tag endogenous LIN-2, LIN-7 and LIN-10 with fluorescent fusion proteins, and we used tissue-specific extrachromosomal arrays to determine the expression and subcellular localization of the LIN-2/7/10 complex with LET-23 EGFR in the VPCs. We found that LIN-7 is the only complex component that colocalizes with LET-23 EGFR on the basolateral membrane of the VPCs; surprisingly, this localization is independent of the LET-23 EGFR C-terminal tail previously shown to bind LIN-7. Importantly, we identified the Golgi as the common site of LIN-2/7/10 complex formation, at which it also colocalizes with LET-23 EGFR. We determined an order of recruitment to the Golgi by which LIN-10 is largely required for the recruitment of LIN-2 and LIN-7 to these compartments. We demonstrate that the LIN-2/7/10 complex forms *in vivo*, but that LIN-2/7 have different expression levels and subcellular localization patterns from LIN-10 in the VPCs and in other tissues, consistent with their stronger interactions. Our results suggest that the LIN-2/7/10 complex forms on Golgi to target LET-23 EGFR to the basolateral membrane of the VPCs rather than functioning as a tether at the basolateral membrane.

## RESULTS

### LIN-2, LIN-7 and LIN-10 localize to cytoplasmic punctae in the VPCs

To understand how the LIN-2/7/10 complex regulates LET-23 EGFR localization, we sought to identify where the LIN-2/7/10 complex forms *in vivo*. We used CRISPR/Cas9 to tag the endogenous 5′ end of the *lin-7* and *lin-10* genes with an mNeonGreen (mNG) fluorophore and a 3xFlag tag, generating the *lin-7(vh51)* (“mNG::LIN-7”) and *lin-10(vh50)* (“mNG::LIN-10”) alleles, respectively (Fig. 1C,G). We also inserted an mKate2 (mK2) fluorophore and a 3xMyc tag to the 3′ end of the *lin-2* gene, generating the *lin-2(vh52)* (“LIN-2::mK2”) allele (Fig. 1E). The gene products are predicted to generate wild-type functional proteins based on the absence of vulval development defects in their respective lines (Table S1). For tissue-specific expression, we generated extrachromosomal transgenes, each under the control of the VPC-specific promoter *lin-31*, of the LIN-7a isoform tagged C-terminally with EGFP (*vhEx87*, “LIN-7a::EGFP”), LIN-2a tagged N-terminally with GFP (*vhEx58*, “GFP::LIN-2a”) and LIN-10a tagged N-terminally with GFP (*vhEx37*, “GFP::LIN-10a”) (Fig. S1A). These transgenes rescued the vulvaless phenotypes of their respective mutants, confirming functionality (Table S2).

**Fig. 2.**
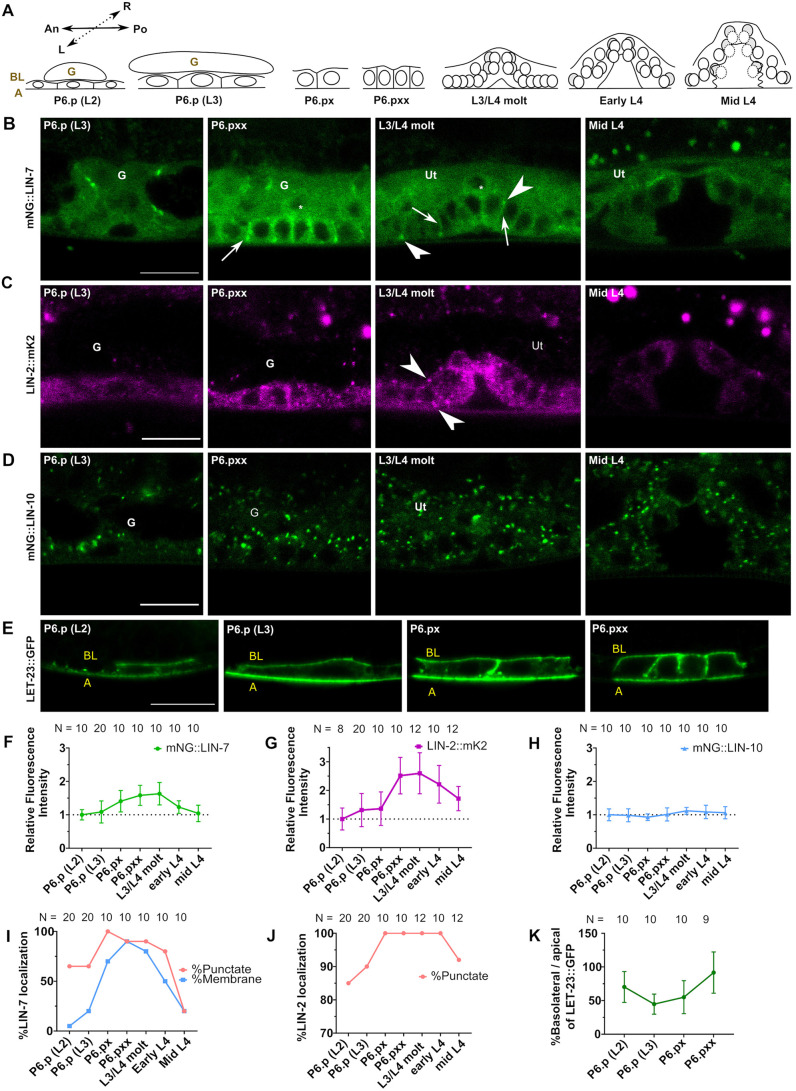
**Expression and localization dynamics of LIN-2/7/10 and LET-23 EGFR across vulva development.** (A) Schematic of the stages of vulval development, from induction (early L2) to mid-morphogenesis (mid L4), used for analysis of fluorescent intensity. Membranes outlining each vulva cell from L3/L4 molt are not shown. An: Anterior. Po: Posterior. L: Left. R: Right. (B) mNG::LIN-7 expression and subcellular localization in P6.p, P6.pxx, L3/L4 molt and mid L4 worms. Arrowheads indicate punctate localization of LIN-7. Arrows indicate membrane localization of LIN-7. Asterisks indicate the nuclei of anchor cells. (C) LIN-2::mK2 expression and subcellular localization in P6.p, P6.pxx, L3/L4 molt and mid L4 worms. Arrowheads indicate punctate localization of LIN-2. (D) mNG::LIN-10 expression and subcellular localization in P6.p, P6.pxx, L3/L4 molt and mid L4 worms. (E) LET-23::GFP (*zhIs035*) expression and localization in P6.p cells from L2 and L3 larvae, P6.px cells, and P6.pxx cells. (F) mNG::LIN-7 cytosolic fluorescent intensity expression analysis from P6.p (L2) to mid-L4. (G) LIN-2::mK2 cytosolic fluorescent intensity analysis from P6.p (L2) to mid L4. (H) mNG::LIN-10 cytosolic fluorescent intensity analysis from P6.p (L2) to mid L4. (I) Percentage of worms imaged with punctate or membrane localization of mNG::LIN-7 from P6.p (L2) to mid L4. (J) Percentage of worms imaged with punctate LIN-2::mK2 localization from P6.p (L2) to mid L4. (K) Average peak basolateral/apical membrane fluorescent intensity ratio analysis of LET-23::GFP from P6.p (L2) to P6.pxx. N, number of animals scored. Data are mean±s.d. An, anterior; A, apical; BL: basolateral; G, gonad; Po, posterior; Ut, uterus. Scale bars: 10 μm.

mNG::LIN-7, LIN-2::mK2 and mNG::LIN-10 were found to be broadly expressed in the worm. In L3 larvae, they were prominently expressed in VPCs and neurons, whereas only LIN-7 and LIN-10 were detectable in the somatic gonad primordium (Fig. S2). In the VPCs, endogenous mNG::LIN-7 was strongly cytosolic, frequently found at punctae, and occasionally localized to basolateral membranes (Fig. 1D, Fig. 2B). C-terminally tagged extrachromosomal LIN-7a::EGFP was similarly cytosolic and occasionally localized to one or two punctae per cell; however, it was not detectable at cell membranes, and instead had nuclear localization (Fig. S1B). The C-terminal PDZ domain has previously been found to be required for cell junction localization of *C. elegans* LIN-7 and mammalian Lin7A in epithelia (Simske et al., 1996; Perego et al., 2000); therefore, placement of the fluorophore at the C-terminus might disrupt recruitment to membranes without compromising overall function. Alternatively, overexpression of LIN-7 from the extrachromosomal array may overwhelm any detectable signal at the plasma membrane.

Endogenous LIN-2::mK2 was also found to have a strong cytosolic signal and to localize to cytoplasmic punctae, but did not have a distinct membrane localization pattern (Fig. 1F, Fig. 2C). N-terminally tagged extrachromosomal GFP::LIN-2a was strongly cytosolic and nuclear. It also localized to punctae, although less frequently: 30% of VPCs imaged had punctate LIN-2 localization (Fig. S1C).

Finally, both endogenous mNG::LIN-10 and extrachromosomal GFP::LIN-10 strongly localized to punctae with a relatively low cytosolic signal (Fig. 1H, Fig. 2D; Fig. S1D), consistent with localization to Golgi ministacks and recycling endosomes previously identified in neuronal and intestinal cells (Rongo et al., 1998; Glodowski et al., 2005; Zhang et al., 2012a). Similar punctate localization for endogenous untagged LIN-10 has been found in immunostaining assays at the P6.pxx four-cell stage (Whitfield et al., 1999). The common pattern of LIN-2, LIN-7 and LIN-10 localization to cytoplasmic punctae in the VPCs suggests that the LIN-2/7/10 complex functions in LET-23 EGFR trafficking.

### Expression of LIN-2, LIN-7 and LET-23 EGFR changes throughout vulval development

**Fig. 3.**
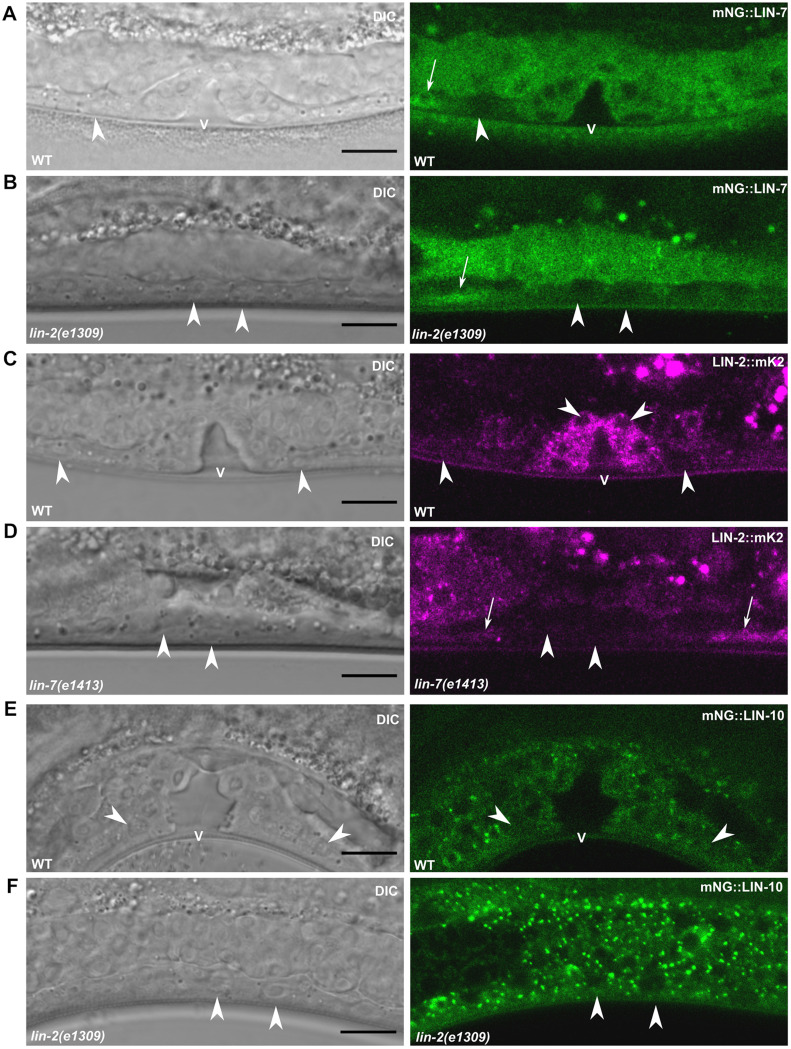
**LIN-2 and LIN-7 expression is reduced in uninduced VPC lineages.** (A,B) mNG::LIN-7 expression in VPC lineages of wild-type (A) and *lin-2* mutant (B) L4 larvae. (C,D) LIN-2::mK2 expression in VPC lineages of wild-type (C) and *lin-7* mutant (D) L4 larvae. (E,F) mNG::LIN-10 expression in VPC lineages of wild-type (E) and *lin-2* mutant (F) L4 larvae. Scale bars: 10 μm. Arrowheads indicate uninduced cells. Arrows indicate a segment of ventral nerve chord in same focal plane as the VPCs. V, vulval lumen.

Many regulators of LET-23 EGFR signaling are differentially expressed or localized before, during and after VPC induction. Expression of LET-23 EGFR itself increases in P6.p cells shortly after signaling activation (Simske et al., 1996). Therefore, we analyzed complex component expression across several stages of vulva development (Fig. 2A). We found that the cytosolic fluorescence intensities associated with LIN-2::mK2 and mNG::LIN-7 increased after P6.p had divided (after cell fate determination) and peaked after all cell divisions had taken place near the L3/L4 molt, then dropped in L4 larvae as vulva morphogenesis progressed (Fig. 2B,C,F,G). Furthermore, their expression was restricted to the induced vulval cell fate lineages: in the uninduced P3.p, P4.p and P8.p cells, and in the uninduced cells of *lin-2(e1309)* or *lin-7(e1413)* null mutants, fluorescence intensity of LIN-2::mK2 and mNG::LIN-7 dropped after one cell division, potentially coincident with cell fusion with Hyp7 (Fig. 3A-D). In addition to changes in fluorescence intensity, mNG::LIN-7 displayed changes in membrane and punctate localization throughout VPC induction. The proportion of worms with distinct mNG::LIN-7 membrane localization increased from 5% of one-cell P6.p at the L2 larval stage (when LET-23 EGFR signaling begins) to 90% of four-cell P6.pxx, then dropped down to 20% in the developing vulva of mid-L4 larvae (Fig. 2I). Although most animals had some faint LIN-7^+^ punctae throughout most of vulval development, the proportion of worms with punctate LIN-7 localization sharply decreased to 20% in mid-L4 vulva. LIN-2 subcellular localization exhibited minor changes in localization: 100% of cells from the two-cell P6.px stage through to early L4, in which vulva morphogenesis occurs, had punctate localization of LIN-2 (Fig. 2J). This proportion decreased to 90% in P6.p cells of L3 larvae and the developing vulva of mid-L4 larva, and was further reduced to 85% of P6.p cells in L2 larvae.

Unlike its complex components, LIN-10 cytosolic fluorescence intensity and localization to punctae did not detectably change throughout vulval development. LIN-10 was expressed evenly in all VPCs and their descendants (Fig. 2D,H), including the non-vulval cell lineages of P3.p, P4.p and P8.p (Fig. 3E,F).

Membrane-bound LET-23 EGFR is localized in a polarized fashion in the VPCs, with stronger fluorescence intensity detected on the apical membrane than on the basolateral membrane (Skorobogata et al., 2014). To look for changes in polarized LET-23 EGFR distribution, we compared the peak fluorescence intensities of an integrated LET-23::GFP transgene (*zhIs035*; Haag et al., 2014) along the basolateral and apical membranes of P6.p (L2 and L3), P6.px and P6.pxx cells. The subsequent cell division generating P6.pxxx is a transverse division along the left/right plane and occurs with the formation of the apical lumen of the vulva (Sternberg and Horvitz, 1986) (Fig. 2A), and as a result, the apical membranes of P6.p lineages face the lumen rather than the ventral side (Sharma-Kishore et al., 1999), obscuring them from imaging and analysis. We found that at the L2 stage, the basolateral/apical fluorescence intensity ratio of LET-23::GFP decreased from ∼75% to below 50% from the L2 to L3 larvae in P6.p cells. After the P6.p cell divided, the ratio increased such that the fluorescent intensity on both membranes was almost even by the P6.pxx stage (Fig. 2E,K). These differences are largely attributed to changes in fluorescence intensity along the apical membrane. These results suggest that the increase in the basolateral/apical ratio of LET-23 EGFR in P6.p cell lineages coincides with the increase in LIN-7 and LIN-2 (but not LIN-10) cytosolic fluorescence intensity, which occurs after cell fate has been established. However, LIN-7 and LIN-2 persist in the secondary (P5.p and P7.p) cell lineages, in which LET-23 EGFR expression is downregulated (Simske et al., 1996). Thus, LIN-2 and LIN-7 expression levels may be uncoupled from LET-23 EGFR signaling.

### LIN-2, LIN-7 and LIN-10 colocalize on cytoplasmic punctae in the VPCs

**Fig. 4.**
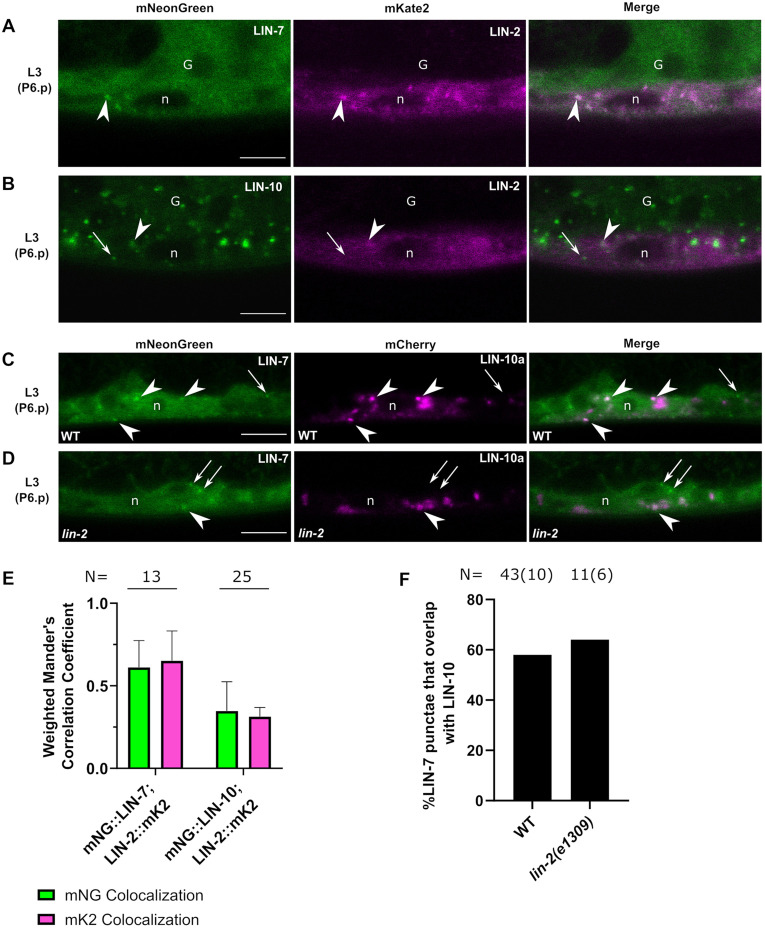
**The LIN-2/7/10 complex colocalizes on cytoplasmic punctae.** (A) mNG::LIN-7 and LIN-2::mK2 colocalize in the cytosol and at punctae in P6.p cells of L3 worms. (B) mNG::LIN-10 and LIN-2::mK2 colocalize at some punctae in P6.p cells of L3 worms. (C,D) Overlap of mNG::LIN-7^+^ with mCherry::LIN-10a punctae in a wild-type (C) and *lin-2* mutant (D) background. (E) Weighted Mander's colocalization coefficients for L3 (P6.p to P6.pxx) worms. N, number of worms analyzed. (F) Quantification of the percentage (on *y*-axis) mNG::LIN-7^+^ punctae that overlap with mCherry::LIN-10a in a wild-type and *lin-2* mutant. N, total number of punctae analyzed, with number of worms in brackets. Data are mean±s.d. Scale bars: 5 μm. Arrowheads indicate colocalizing punctae. Arrows indicate non-colocalizing punctae. G, L3 gonad; WT, Wild type.

To determine whether the LIN-2/7/10 complex colocalizes on cytoplasmic punctae, we crossed both *lin-7(vh51)* and *lin-10(vh50)* with *lin-2(vh52)*. To capture the dynamic range of LIN-7 localization patterns, and to account for the low fluorescence intensity of LIN-7 and LIN-2 at the time of vulval cell fate induction (early L2), we performed colocalization analyses in worms ranging from late L2 to late L3 (P6.pxx). We found that interacting partners LIN-7 and LIN-2 colocalized strongly in the cytosol and at cytoplasmic punctae of the VPCs (Fig. 4A), although LIN-2 is frequently localized to punctae without LIN-7. Mander's correlation coefficients revealed moderately strong colocalization between LIN-2 and LIN-7 (Fig. 4E).

There was some overlap between LIN-2 and LIN-10 at cytoplasmic punctae; however, they were frequently localized to separate compartments (Fig. 4B). Mander's colocalization coefficients revealed relatively weak colocalization between LIN-10 and LIN-2 (Fig. 4E). Similar levels of colocalization were seen in the developing vulvas of L4 worms (Fig. S3A,B,E), suggesting that these colocalization patterns are stable through development and unlikely to be signaling dependent.

To test for overlap between LIN-7 and LIN-10, we crossed *lin-7(vh51)* with an extrachromosomal mCherry-tagged LIN-10a transgenic line (*vhEx63*) (mCh::LIN-10), and found that 58% of LIN-7^+^ punctae overlapped with LIN-10^+^ punctae (Fig. 4C,F). LIN-2, which bridges LIN-7 and LIN-10 in the complex, was not required for LIN-7 punctae to overlap with LIN-10 (Fig. 4D,F). However, there were far fewer LIN-7 punctae in a *lin-2(e1309)* mutant compared with wild type, as described below in Fig. 8.

### LET-23 EGFR colocalizes with LIN-7 at the plasma membrane and with LIN-10 at cytoplasmic punctae in the VPCs

**Fig. 5.**
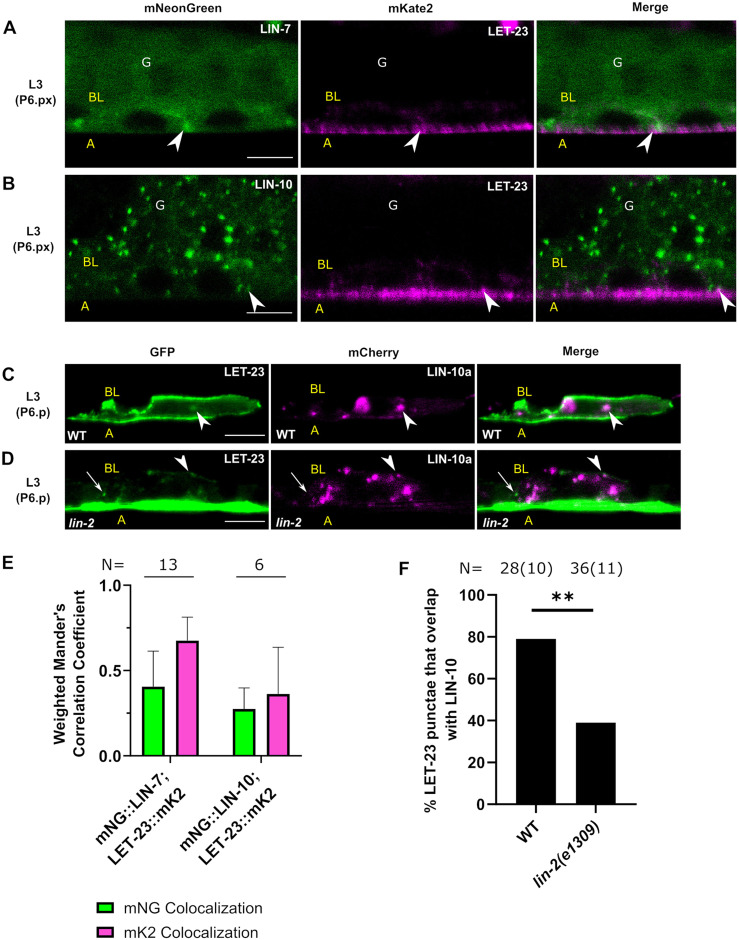
**LET-23 EGFR colocalizes with LIN-7 at basolateral membranes and with LIN-10 at punctae.** (A) mNG::LIN-7 and LET-23::mK2 (*re202*) overlap at basolateral membranes in L3 (P6.px) worms. (B) mNG::LIN-10 and LET-23::mK2 infrequently overlap in L3 (P6.px) worms. (C,D) Overlap of LET-23::GFP^+^ punctae with mCherry::LIN-10a^+^ punctae in wild type (C) and a *lin-2* mutant (D). (E) Weighted Mander's colocalization coefficients for L3 (P6.p to P6.pxx) worms. N, number of worms analyzed. (F) Quantification of LET-23::GFP^+^ punctae that overlap with mCh::LIN-10a^+^ puncta, as shown in C and D, in wild type and a *lin-2* mutant. N, total number of punctae analyzed, with number of worms in brackets. Data are mean±s.d. **P<0.01 (Fisher's exact test). Scale bars: 5 μm. Arrowheads indicate colocalizing puncta/membranes. Arrows indicate non-colocalizing punctae. A, Apical; BL, Basolateral; G, L3 gonad; WT, Wild type.

To identify where the LIN-2/7/10 complex might interact with LET-23 EGFR, we crossed *lin-7(vh51)* and *lin-10(vh50)* with a strain expressing endogenously tagged LET-23::mKate2::3xFlag [*let-23(re202)*; “LET-23::mK2”; generated by CRISPR/Cas9; generously provided by T. Duong and D. Reiner, Texas A&M University, College Station, TX, USA]. LET-23::mK2 localized to the basolateral and apical membrane domains of the VPCs (Fig. 5A,B), as has been described previously for endogenous LET-23 EGFR and other LET-23 EGFR reporters (Haag et al., 2014; Skorobogata et al., 2014; Whitfield et al., 1999). The fluorophore was placed upstream of the PDZ interaction motif on the C-terminal end to preserve the interaction with LIN-7, similar to other functional LET-23::GFP transgenes (Haag et al., 2014). However, basolateral receptor localization was often undetectable until the two-cell P6.px stage (past the point of cell fate determination), and the presence of mild and infrequent vulval abnormalities suggested that the modifications made to the endogenous *let-23* gene locus caused a minor disruption to its regular function (Table S1).

We found that LET-23::mK2 and mNG::LIN-7 overlapped at the basolateral plasma membrane in L3 larvae (from P6.p to P6.pxx) (Fig. 5A). This overlap was more apparent in the differentiated vulval cells (L4) (Fig. S3C). This suggests that LIN-7 interacts with the receptor at the cell periphery. This interaction might happen in the absence of LIN-2 because LIN-2::mK2 did not reveal any apparent membrane localization. Mander's correlation coefficients showed that mNG::LIN-7 colocalizes weakly with LET-23::mK2; however, the receptor colocalized with mNG::LIN-7 relatively strongly (Fig. 5E; Fig. S3F).

On the other hand, there was minimal colocalization between LET-23::mK2 and mNG::LIN-10 (Fig. 5B,E; Fig. S3D,F), although LIN-10 did occasionally overlap with LET-23 EGFR at the cell periphery (Fig. 5B). We previously found that LET-23::GFP typically localizes to a few small cytoplasmic punctae in most P6.p and P6.px cells (Skorobogata et al., 2016). LET-23::mK2 fluorescence intensity was too low to detect cytosolic puncta; therefore, to determine whether LIN-10 might colocalize with LET-23 EGFR intracellularly, we crossed the LET-23::GFP integrated transgene with extrachromosomal mCh::LIN-10a and found that most cytosolic LET-23::GFP^+^ punctae overlapped with LIN-10 (Fig. 5C,F). This overlap was significantly decreased in a *lin-2* mutant, though not eliminated entirely (Fig. 5D,F), consistent with LIN-2 promoting the association between LIN-10 and LET-23 EGFR.

### LIN-2 and LIN-7 colocalize in neurons, but not with LIN-10 and LET-23

**Fig. 6.**
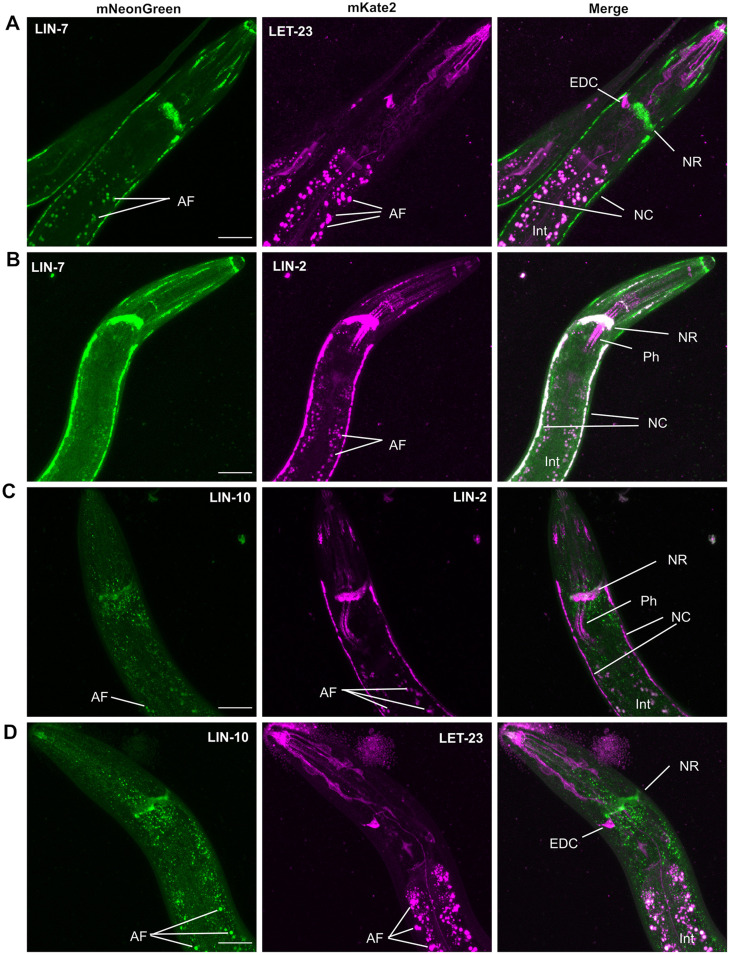
**LIN-2 and LIN-7, but not LIN-10 or LET-23 EGFR, colocalize in neurons.** (A-D) Three-dimensional *z*-stack maximum intensity projections of the anterior half of L3 (A-C) and L4 (D) larval worms. (A) mNG::LIN-7 and LET-23::mK2 EGFR were both expressed in several tissues in the head of *C. elegans* but do not colocalize in overlap. (B) mNG::LIN-7 and LIN-2::mK2 colocalized strongly in neuronal tissues. (C) LIN-2::mK2 and mNG::LIN-10 overlapped minimally in the nerve ring. (D) mNG::LIN-10 and LET-23::mK2 EGFR had distinct expression patterns in the head. Scale bars: 20 μm. AF, autofluorescence; EDC, excretory duct cell; Int, intestine; NC, nerve chords; NR, neural ring; Ph, Pharynx.

Endogenously tagged LIN-2, LIN-7, LIN-10 and LET-23 EGFR allow for the analysis of their localization and expression patterns in other tissues. We found that all four proteins are expressed in neurons and sensory tissue in the head, and along the ventral and dorsal nerve chords (Fig. 6; Fig. S2). The intestine is prone to a high degree of autofluorescence and was excluded from the initial analysis. Whereas LET-23 EGFR and LIN-7 overlapped minimally in the head (Fig. 6A), LIN-2 and LIN-7 colocalized strongly in the neural ring, and the ventral and dorsal nerve chords (Fig. 6B). Of note, we observed that LIN-2 was more strongly expressed in the isthmus of the pharynx than LIN-7 (Fig. 6B). In contrast, LIN-7 was more strongly expressed in the gonad and uterus than LIN-2 (see Figs 1–5; Fig. S2A,B). LIN-10 overlapped minimally with LIN-2 in the neural ring and nerve chords (Fig. 6C), and shared very little overlap with LET-23 EGFR in other neural tissues in the head of the worm (Fig. 6D). LET-23::mK2 was strongly expressed in the excretory duct cell (Fig. 6A,D) in which it signals through the LET-60 Ras/MPK-1 ERK pathway to regulate excretory duct cell development (Abdus-Saboor et al., 2011; Yochem et al., 1997). Loss-of-function mutations in *let-23*, but not in *lin-2/7/10* complex components, yield a larval lethal phenotype associated with the loss of the excretory duct cell (Ferguson and Horvitz, 1985). The LIN-2/7/10 complex components were not observed in the excretory duct cell, consistent with them not being required for LET-23 EGFR signaling in that cell.

### LIN-2::mK2 interacts strongly with mNG::LIN-7 and minimally with mNG::LIN-10 *in vivo*

The specific interactions between LIN-2 and LIN-7, and between LIN-2 and LIN-10 have been tested by yeast two-hybrid assay, and complex formation has been confirmed by co-immunoprecipitation of the *C. elegans* proteins expressed exogenously in *Drosophila* S2 cells (Kaech et al., 1998). These interactions have been shown to be evolutionarily conserved, and the complex has been shown to form in mammalian neurons by co-immunoprecipitation and *in vitro* pull-down assays (Butz et al., 1998; Borg et al., 1998; Becamel et al., 2002; Leonoudakis et al., 2004). It has not yet been shown that the complex forms *in vivo* in *C. elegans*, and the disparity in the observed localization patterns (Figs 2, 4, and 6) call into question the extent of these interactions *in vivo*.

**Fig. 7.**
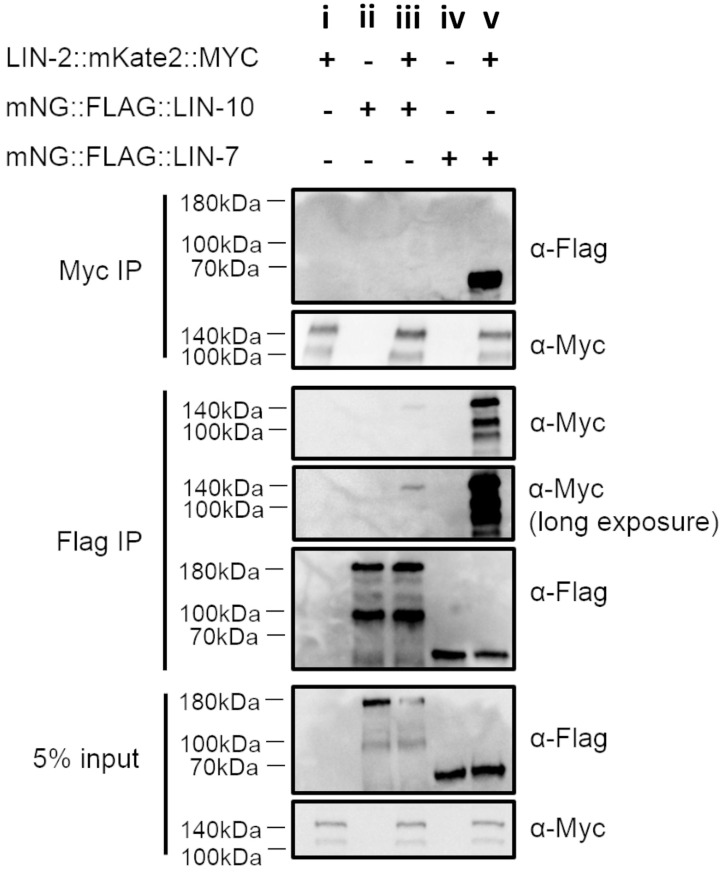
**LIN-2, LIN-7 and LIN-10 interact *in vivo*.** Co-immunoprecipitation assays using whole worm lysate from worms expressing (i) LIN-2::mK2::3xMyc, (ii) mNG::3xFlag::LIN-10, (iii) both LIN-2::mK::3xMyc and mNG::3xFlag::LIN-10, (iv) mNG::3xFlag::LIN-7 and (v) both LIN-2::mK::3xMyc and mNG::3xFlag::LIN-7.

To test whether the proteins interact *in vivo*, we performed a co-immunoprecipitation assay using whole worm lysates (Fig. 7). On a western blot, we found that mNG::3xFlag::LIN-10 yielded two bands. The larger band at ∼180 kDa is expected to include the three known isoforms of LIN-10 (a, b and c), and is about 45 kDa larger than anticipated for the fusion of LIN-10 with a fluorophore. A similar size shift has been previously reported using antibodies for endogenous LIN-10, and could be a result of extensive post-translational modifications (Whitfield et al., 1999). A second smaller band was also observed for LIN-10, as expected based on previously published work, and may represent an uncharacterized splice variant or proteolytic cleavage (Whitfield et al., 1999). Blotting for mNG::3xFlag::LIN-7 yielded the expected single band at ∼65 kDa (expected to represent both a and b isoforms), and LIN-2::mK::3xMyc yielded the expected two bands at 140 kDa and 100 kDa, representing the full-length a isoform and shorter b isoform, respectively (Fig. 7).

The full-length LIN-2a isoform co-immunoprecipitated with both LIN-7 and LIN-10 from whole animal lysates. On the other hand, the truncated LIN-2b isoform lacking the N-terminal CamKII domain only precipitated with LIN-7, consistent with this domain being required for interaction with LIN-10 (Borg et al., 1998; Kaech et al., 1998). Furthermore, although LIN-7 and LIN-2 co-immunoprecipitated strongly together, LIN-2 co-immunoprecipitated weakly with LIN-10, and no LIN-10 was recovered when purifying LIN-2 from lysates (Fig. 7). These results and the colocalization analysis suggest LIN-2 and LIN-7 interact stably in several tissues, whereas LIN-2 and LIN-10 interact minimally. These results, along with the colocalization data, suggest that LIN-2 and LIN-7 might exist as a subcomplex.

### LIN-10 recruits LIN-2 and LIN-7 to a subset of cytosolic punctae

**Fig. 8.**
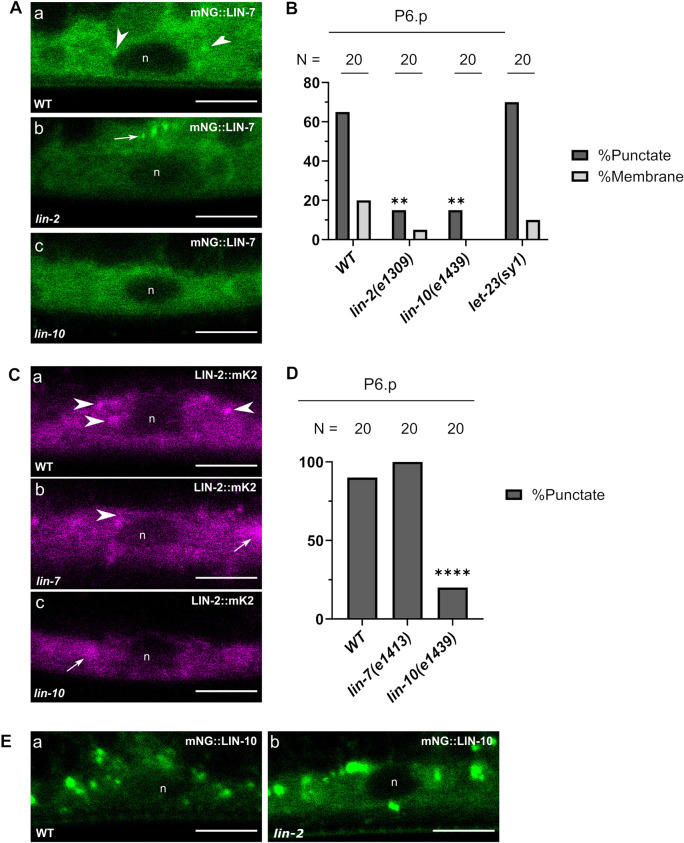
**Punctate localization of LIN-2 and LIN-7 are complex dependent.** (A) mNG::LIN-7 localization to punctae (Aa) is decreased in *lin-2* (Ab) *lin-10* (Ac) mutants. Arrowheads indicate punctate localization of mNG::LIN-7 in P6.p. Arrow indicates punctate localization of mNG::LIN-7 in the anchor cell. (B) Quantification of mNG::LIN-7 localization patterns in *lin-2*, *lin-10* and *let-23(sy1)* mutants. (C) Analysis of endogenously tagged LIN-2::mK2 localization to punctae (Ca) is unchanged in a *lin-7* mutant (Cb), but decreases in a *lin-10* mutant (Cc). Arrowheads indicate punctate localization of LIN-2::mK2 in P6.p. Arrows indicate fluorescent expression in the ventral nerve chord in same focal plane as VPCs. (D) Quantification of LIN-2::mK2 localization patterns in *lin-7* and *lin-10* mutants. (E) Localization of mNG::LIN-10 to punctae (Ea) is unchanged in a *lin-2* mutant (Eb). **P<0.01, ****P<0.0001 (Fisher's Exact Test compared with wild type). n, nucleus; N, numbers of animals scored. Scale bars: 5 μm.

Given the strong colocalization between LIN-2 and LIN-7, and their dependency for localization in some mammalian epithelial cells (Straight et al., 2000; Lozovatsky et al., 2009), we tested the interdependency among LIN-2, LIN-7 and LIN-10 for their localization in the VPCs. Because mNG::LIN-7 and LIN-2::mK2 expression was lost in uninduced cells (Fig. 3), we limited our analysis to P6.p cells in late L2 and L3 worms (before cell fate determination; Fig. 8), and to L4 worms with partial or full vulval development (in which LIN-7 and LIN-2 are expressed; Fig. S4A,B). We found that LIN-7 punctate localization is largely LIN-2- and LIN-10-dependent. mNG::LIN-7, which localizes to punctae in 65% of L3 worms (P6.p) and 50% of all L4 worms (pooled for both early- and mid-L4), was localized to punctae in only 15% of P6.p cells and 10% of L4 worms with partial or full vulval development in *lin-2(e1309)* null mutants (Fig. 8A,B; Fig. S4A). In *lin-10(e1439)* null mutants, punctate localization of LIN-7 was similarly decreased to 15% of P6.p cells and 15% of L4 worms with partial or full vulval development (Fig. 8A,B; Fig. S4A). LIN-7 basolateral membrane localization, on the other hand, was not significantly altered in *lin-2(e1309)* and *lin-10(e1439)* mutants (Fig. 8B; Fig. S4A).

We found that LIN-2 localization to cytoplasmic punctae was in turn partly LIN-10 dependent, and LIN-7 independent. The predominately punctate LIN-2::mK2 became exclusively cytoplasmic in 80% of P6.p cells in *lin-10(e1439)* null mutants (Fig. 8C,D). The dependency was less pronounced, but still evident at the L4 stage (Fig. S4B). Consistently, GFP::LIN-2a, expressed from an extrachromosomal transgene, localized to faint punctae in ∼30% of the VPCs imaged, was almost completely cytosolic in a *lin-10(e1439)* mutant (Fig. S4C,D). Punctate localization of LIN-2::mK2 was unaltered in a *lin-7(e1413)* mutant in the VPCs (Fig. 8C,D; Fig. S4B).

On the other hand, we found that both endogenously tagged mNG::LIN-10 and extrachromosomal GFP::LIN-10a punctate localization was not altered with loss-of-function mutations in *lin-2* and *lin-7* (Fig. 8E; Fig. S4E), suggesting that LIN-10 maintains its localization pattern independently of complex formation. Together, these data demonstrate that LIN-10 recruits LIN-2 and LIN-7 to cytoplasmic punctae, and are consistent with LIN-2 bridging an interaction between LIN-10 and LIN-7.

Finally, we found that both LIN-10 and LIN-7 localization was LET-23 EGFR independent. The *sy1* allele of the *let-23 egfr* gene contains an early stop codon that truncates the last six amino acids, resulting in the loss of LET-23 EGFR protein interaction with the PDZ domain of LIN-7 (Aroian and Sternberg, 1991; Aroian et al., 1994; Kaech et al., 1998). Although the *sy1* mutant LET-23 is otherwise signaling-competent in other LET-23-dependent developmental events in the animal, it is localized exclusively to the apical membrane in the VPCs and cannot induce the vulval cell fate (Kaech et al., 1998). LIN-7 was also not mislocalized to apical membranes in a *let-23(sy1)* mutant; instead, LIN-7 remained associated to basolateral membranes in 20% of P6.p cells analyzed, and retained its punctate localization in 70% of cells (Fig. 8B). Extrachromosomal GFP::LIN-10a localization was also unaltered in a *let-23(sy1)* mutant (Fig. S4E). This suggests that LIN-7 and LIN-10 are appropriately localized to their respective subcellular domains independently of an association with the LET-23 EGFR C-terminal PDZ interaction motif.

### LIN-10 and LIN-7 localize to the Golgi

**Fig. 9.**
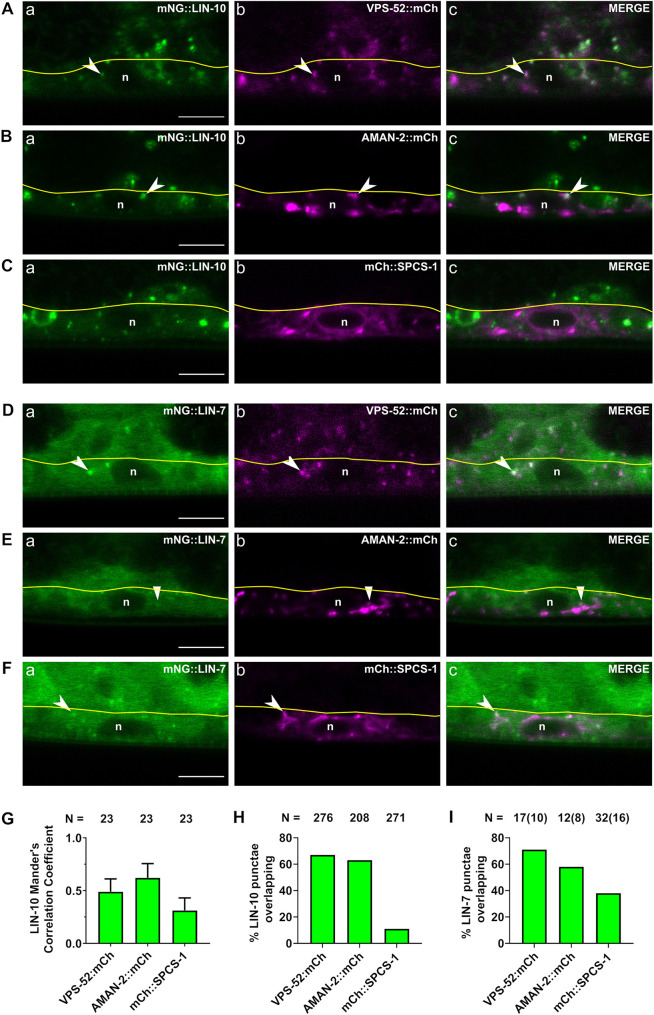
**LIN-10 and LIN-7 localize to the Golgi.** (A-C) mNG::LIN-10 punctae overlap with Golgi and recycling endosome marker VPS-52 (*qbcSi01*) (A), and with trans-Golgi marker AMAN-2 (B), but not with ER marker SPCS-1 (C). (D-F) mNG::LIN-7 punctae overlap with Golgi and recycling endosome marker VPS-52 (*qbcSi01*) (D) and with trans-Golgi marker AMAN-2 (E), and partly with ER marker SPCS-1 (F). (G) Correlation coefficient of LIN-10 with VPS-52, AMAN-2 and SPCS-1. N, number of animals scored. (H) Percentage of mNG::LIN-10^+^ punctae that overlap with VPS-52, AMAN-2 and SPCS-1. (I) Percentage of mNG::LIN-7^+^ punctae that overlap with VPS-52, AMAN-2 and SPCS-1. N, number of punctae scored (H,I), with number of animals in brackets. n, nucleus. Arrowheads indicate colocalizing punctae in VPCs. Yellow line indicates the basal lamina to demarcate the VPC from the overlying gonad. Scale bars: 5 μm.

LIN-10 has been shown to localize to Golgi mini-stacks and recycling endosomes in *C. elegans* neurons and intestinal cells (Whitfield et al., 1999; Glodowski et al., 2005; Zhang et al., 2012a), and the homologous APBA1-3 proteins localize to the trans*-*Golgi network in mammalian neurons (Stricker and Huganir, 2003; Borg et al., 1999). mLin7 has been implicated in recycling internalized cargo back to the plasma membrane (Shelly et al., 2003; Straight et al., 2001), but has not previously been localized to cytoplasmic compartments. To determine whether the punctate localization of LIN-10 and LIN-7 in the VPCs corresponds to the Golgi, we tested for colocalization with AMAN-2, a marker of the Golgi (Paschinger et al., 2006; Velasco et al., 1993), and VPS-52, a shared subunit of the trans*-*Golgi-associated GARP complex and recycling endosome-associated EARP complex (Luo et al., 2011; Conibear and Stevens, 2000; Liewen et al., 2005; Schindler et al., 2015). As a control for specificity, we tested for colocalization with SPCS-1 (SP12), a signal peptidase complex protein found in the endoplasmic reticulum (Haag et al., 2020; Rolls et al., 2002). LIN-10^+^ punctae regularly overlapped with both AMAN-2 and VPS-52 in the VPCs of L2 and L3 (P6.p to P6.px) larvae, and minimally overlapped with SPCS-1 (Fig. 9A-C,H). This is supported by Mander's correlation coefficients, which reveal moderate correlation of LIN-10 with VPS-52 and AMAN-2 (0.49 and 0.62, respectively), and low correlation with SPCS-1 (0.13) (Fig. 9G). LIN-7^+^ punctae also overlapped with AMAN-2 and VPS-52 at a comparable frequency to LIN-10, although LIN-7 did not localize to punctae as frequently as LIN-10 overall (Fig. 9D,E,I). LIN-7 punctae had a greater frequency of overlap with SPCS-1 than LIN-10 (Fig. 9H,I). If LIN-10 and LIN-7 localized to both recycling endosomes and the Golgi, we would expect a greater degree of overlap with VPS-52 than with AMAN-2, as VPS-52 would represent a larger pool of their subcellular localizations. Instead, we found similar overlap with VPS-52 and with AMAN-2, consistent with LIN-10 and LIN-7 predominately localizing to the Golgi. Given the strong colocalization between LIN-2 and LIN-7, these observations suggest that the LIN-2/7/10 complex localizes to the Golgi in the VPCs.

## DISCUSSION

The evolutionarily conserved LIN-2/7/10 complex has been well characterized biochemically and genetically, but where and how it regulates LET-23 EGFR localization to the basolateral membrane of the VPCs was unknown. Here, we tagged each endogenous component of the LIN-2/7/10 complex and systematically characterized their expression and subcellular localization, their colocalization with each other and with LET-23 EGFR, and their interdependence for localization. LIN-7 was the only complex component observed to localize with LET-23 EGFR at the basolateral membrane of the VPCs. We identified the Golgi as the common site of LET-23 EGFR localization with the LIN-2/7/10 complex. We demonstrated that LIN-10 is largely required for the recruitment of LIN-2 and LIN-7 to the Golgi and point toward a role for the LIN-2/7/10 complex in basolateral membrane targeting of intracellular LET-23 EGFR.

### Polarized distribution of LET-23 EGFR in the VPCs

LET-23 EGFR localizes to the basolateral and apical membranes of the VPCs. Despite stronger localization at the apical membrane, it is the basolateral membrane localization that is thought to mediate inductive signaling as the ligand secreting cell is adjacent to the basolateral membrane, and mutations in *lin-2*, *lin-7* or *lin-10* result in loss of basolateral LET-23 EGFR and a strong Vul phenotype. Apical localization might represent a default trafficking pathway for LET-23 EGFR that requires an interaction with the LIN-2/7/10 complex to overcome.

Apical localization of LET-23 EGFR is mediated, at least in part, by an Arf GTPase and AP-1 clathrin adaptor complex pathway. Suppression of this pathway results in increased basolateral membrane localization and increased LET-23 EGFR signaling, leading to a multivulva (Muv) phenotype (Skorobogata et al., 2014; Shim et al., 2000). Thus, LET-23 EGFR may be subjected to competing signals that regulate the levels on the basolateral membrane to maintain the fidelity of vulva induction.

The levels of LET-23 EGFR at the basolateral membrane that are required for normal signaling are very low. The *lin-2* Vul phenotype is easily suppressed by the loss of negative regulators of the LET-23 EGFR signaling cascade that are not expected to regulate LET-23 EGFR localization (e.g. Hajnal et al., 1997; Berset et al., 2005), indicating that there is still some signal activation in a *lin-2* mutant; however, signaling is below the threshold for the induction of vulval cell fates. Increased expression of LET-23 EGFR through the introduction of the low copy transgenes (*zhIs035* and *zhIs038*) rescues the *lin-2* Vul phenotype, even when little or no GFP is visible at the basolateral membrane (Skorobogata et al., 2014). Although the endogenously tagged LET-23::mK2 is barely detectable on the basolateral membrane at the time of VPC cell fate induction, these worms have largely normal vulva development. Therefore, the amount of LET-23 needed on the basolateral membrane for vulva induction is below detection using our current reporters.

### LIN-2/7/10 localizes with LET-23 EGFR at the Golgi

Knowing where the LIN-2/7/10 complex forms and colocalizes with LET-23 EGFR provides insight into how the complex might function. Although LIN-7 does localize to the basolateral membrane with LET-23 EGFR, the common site of localization of the LIN-2/7/10 complex with LET-23 EGFR turns out to be intracellular compartments. Owing to its complex-independent role in regulating trafficking in interneurons and its localization to the Golgi and recycling endosomes, LIN-10 is the most studied of the complex in *C. elegans* (Glodowski et al., 2005; Rongo et al., 1998; Zhang et al., 2012a; Whitfield et al., 1999). Thus, it was not surprising that LIN-10 showed strong localization to intracellular compartments that overlap with markers for the trans-Golgi network and recycling endosomes (AMAN-2 and VPS-52). We did not observe LIN-10 localization at the basolateral membrane, except for occasional punctae near the cell periphery, and LIN-10 overlapped with LET-23 EGFR on intracellular compartments, suggesting that LIN-10 promotes basolateral secretion or recycling of LET-23 EGFR to the plasma membrane from the Golgi.

In contrast to LIN-10, LIN-2 and LIN-7 are highly cytosolic with less pronounced localization to cytoplasmic punctae that partly overlap with LIN-10 in the VPCs. LIN-7^+^ punctae overlapped substantially with Golgi and recycling endosome markers, similar to LIN-10. Localization to intracellular punctae and the Golgi is a novel finding for LIN-7 and its orthologs, and has been infrequently reported for LIN-2 and mammalian CASK (Borg et al., 1999; Tong et al., 2015). LIN-2 colocalizes with both LIN-7 and LIN-10 at cytoplasmic punctae in the VPCs, indicating that the Golgi is the main site of complex formation.

Furthermore, we identified a hierarchy of recruitment whereby LIN-10 recruits LIN-2, which in turn recruits LIN-7 to the Golgi. These data are also consistent with studies showing that LIN-2 bridges LIN-7 and LIN-10 (Kaech et al., 1998). Although we saw some LIN-7 overlapping with LIN-10 in the absence of LIN-2, the number of LIN-7 punctae was very low, suggesting that LIN-2 may be needed to stabilize the association of LIN-7 with LIN-10^+^ compartments.

### LIN-2/7 subcomplex

Although the complex colocalizes at the Golgi, LIN-2 and LIN-7 both localize to compartments without LIN-10, and their strong colocalization with each other suggests that LIN-2 and LIN-7 may form a subcomplex. This is supported by our *in vivo* interaction assay. Interestingly, the LIN-2b isoform that interacts with LIN-7, but not LIN-10, could also account for LIN-10^−^ compartments. Alternatively, LIN-2/7-only vesicles could represent recycling or endosomal intermediates that transit through LIN-10^+^ Golgi compartments. Mammalian Lin7 and *C. elegans* LIN-2 have been implicated in endocytic recycling (Straight et al., 2001; Shelly et al., 2003; Stetak et al., 2006), although localization to endocytic membranes has not previously been demonstrated. In the VPCs, LIN-2 interacts with EPS-8 to inhibit internalization of LET-23 EGFR at the basolateral membrane or to promote LET-23 EGFR recycling (Stetak et al., 2006). Loss of EPS-8 or mutation of the first L27 domain of LIN-2, which binds EPS-8 (LIN-7 binds the second L27 domain), results in LET-23 EGFR accumulation in early endosomes. Structural analyses on the formation of stable heterotrimer complexes formed by proteins like LIN-2 that host two L27 domains (Zhang et al., 2012b; Yang et al., 2010) suggest that LIN-2 and LIN-7 subcompartments may also include EPS-8.

### LIN-7 at the basolateral membrane

We found that LIN-7 uniquely localizes to the basolateral membrane, where it can partly colocalize with LET-23 EGFR, consistent with reported localization for mammalian Lin7 (Straight et al., 2000). Although we did not detect any enrichment of LIN-2 or LIN-10 at the plasma membrane, we cannot rule out the possibility that low levels of LIN-2 or LIN-10 function with LIN-7 at the basolateral membrane, or that the fluorescent tag interferes with localization, as may be the case with C-terminally tagged LIN-7a::EGFP. This observation may indicate that LIN-7 alone interacts with the receptor at the plasma membrane, in addition to forming a complex with LIN-2 and LIN-10. Supporting this idea of a complex independent function, overexpression of LIN-7 is sufficient to rescue vulva development in *lin-2* and *lin-10* mutants (Gauthier and Rocheleau, 2021). Interestingly, the PDZ-interaction motif of LET-23 EGFR is not required for LIN-7 localization to either punctae or the basolateral membrane, suggesting that LIN-7 localization is independent of LET-23 EGFR. Alternatively, there could be a second point of interaction between LIN-7 and LET-23 EGFR, as has been found with their mammalian orthologs (Shelly et al., 2003), that may regulate LIN-7 localization.

In *Drosophila* and mammals, LIN-7 is a core component of the Crumbs complex, which localizes on the apical membrane adjacent to cell junctions to establish and maintain epithelial cell polarity (Bulgakova and Knust, 2009; Bachmann et al., 2004; Roh et al., 2002). Crumbs also interacts with moesin in flies (Médina et al., 2002; Speck et al., 2003), a member of the Ezrin/Radixin/Moesin (ERM) family of apical membrane-associated proteins required for the maintenance of epithelial polarity (Van Furden et al., 2004; Gobel et al., 2004). In the VPCs, the moesin ortholog ERM-1 instead localizes to the basolateral membrane in which it interacts with LET-23 EGFR independent of the LIN-2/7/10 complex. ERM-1 restricts lateral movement of LET-23 EGFR within the basolateral membrane, possibly via interactions with the actin cytoskeleton, and is thought to maintain a pool of inactive LET-23 EGFR to limit internalization (Haag et al., 2014). It would be interesting to determine whether LIN-7 could function or compete with ERM-1 on the basolateral membrane to regulate LET-23 EGFR localization, and whether the Crumbs complex might mediate the function of these two proteins.

### LIN-2 and LIN-7 are dynamically expressed during vulval development

We found that LIN-2 and LIN-7 expression levels increase in induced vulval cells during development while also decreasing in the VPCs that do not adopt vulval fates. On the other hand, LIN-10 expression remains stable throughout vulval development and in uninduced VPCs. LET-23 EGFR expression has previously been found to be upregulated in primary cell lineages (P6.p) and downregulated in secondary cell lineages (P5.p and P7.p), and MPK-1 ERK activation is amplified in primary lineages (Kaech et al., 1998; Stetak et al., 2006; de La Cova et al., 2017); however, LIN-2 and LIN-7 expression persists in both cell types. Therefore, LIN-2 and LIN-7 expression is unlikely to be regulated by LET-23 EGFR signaling. The apparent loss of LIN-2 and LIN-7 expression in uninduced VPCs might be due to diffusion of LIN-2 and LIN-7 fusion proteins across the syncytial tissue of the hypodermis following fusion of uninduced VPCs with Hyp7. The upregulation of LIN-2 and LIN-7 expression during vulval morphogenesis suggests that these proteins may have other uncharacterized roles in vulval development, as suggested by previously reported non-Vul egg-laying defective phenotypes seen in hypomorphic *lin-2* and *lin-7* mutants (Ferguson and Horvitz, 1985).

In summary, we present the first comprehensive analysis of LIN-2, LIN-7 and LIN-10 localization in the VPCs during LET-23 EGFR-mediated vulva induction and development. Our results are consistent with a model in which LIN-10 recruits LIN-2 and LIN-7 to the Golgi to promote basolateral targeting of LET-23 EGFR via the biosynthetic secretory pathway, endosome recycling via the trans-Golgi network or both. Our results also suggest that LIN-7 could have additional roles at the plasma membrane. This study offers new insights into how this evolutionarily conserved complex of PDZ domain scaffolding proteins is systematically recruited to the Golgi to promote membrane trafficking.

## MATERIALS AND METHODS

### Strains and maintenance

*C. elegans* strains were maintained as described previously (Brenner, 1974). Worms were grown on *E. coli* HB101 at 20°C and all strains were derived from the N2 wild-type strain. A complete list of strains can be found in Table S3.

### Generating endogenously tagged transgenic strains by CRISPR/Cas9

Primers used for cloning and genotyping are listed in Table S4. mNeonGreen::3xFlag::LIN-10 [*lin-10(vh50)*], mNeonGreen::3xFlag::LIN-7 [*lin-7(vh51)*] and LIN-2::3xMyc::mKate2 [*lin-2(vh52)*] were generated by CRISPR/Cas9 cloning following the self-excising cassette method (Dickinson et al., 2015). The following guide RNA sequences were used: 5′-ACCATGAACAATTCTGTTGC-3′ for *lin-10*; 5′-TTCCAGATGGATAACCCGGA-3′ for *lin-7*; and 5′-GATCAGTAGACCCAAGTGAC-3′ for *lin-2*. Guide RNAs were designed using a prediction software developed by Dr Feng Zhang's lab at the Massachusetts Institute of Technology (crispr.mit.edu). Newly generated alleles were confirmed by PCR and sequencing, and gene products were validated by western blotting, which ran at the expected sizes (Fig. 7).

### Generation of extrachromosomal array lines

Extrachromosomal *vhEx37* and *vhEx58* were cloned by inserting the open reading frames of *lin-10a* and *lin-2a* (amplified from wild-type cDNA, Table S4), respectively, downstream of codon-optimized GFP into a vector with a *lin-31* promoter and an *unc-54* 3′ untranslated region provided by Dr Chris Rongo (Rutgers University, Piscataway, NJ, USA). Extrachromosomal *vhEx63* was cloned by replacing the GFP of *vhEx37* with mCherry. Extrachromosomal *vhEx87* was cloned by inserting *lin-7a* open reading frame and EGFP into a p255 expression vector under the control of a *lin-31* promoter (Skorobogata et al., 2014). All expression vectors (40 ng/µl) were injected with a *pttx-3::gfp* co-injection marker (80 ng/µl) following established protocols (Mello et al., 1991; Hobert et al., 1997).

### Microscopy and image analysis

Epifluorescent (black and white) images were acquired on a Zeiss Axio A1 Imager. Confocal (colorized) images were acquired using an LSM780 laser-scanning confocal microscope (Zeiss). Live animal imaging was performed as described previously (Sulston and Horvitz, 1977).

Punctate or membrane localization of LIN-2, -7, -10 and LET-23 were determined by visual inspection and confirmed by comparing fluorescence intensity of the region of interest to the background cytosolic fluorescence intensity. Peaks in fluorescence spanning the punctae or membrane region of at least twice the intensity of cytosolic fluorescence were categorized as punctate or membrane-associated.

Cytosolic fluorescence intensity was measured by sampling three 1 µm^2^ cytosolic regions (using ImageJ) in primary cell lineages free of any apparent punctae to achieve consistent measurements. Three 1 µm^2^ regions of background fluorescence were also randomly sampled from regions of the image with no worm present. The difference between the average cytosolic fluorescence intensity and the average background fluorescence was used as a measurement of fluorescence intensity for each image.

Polarized membrane distribution of LET-23 EGFR was analyzed as described previously by drawing 20-pixel-wide lines across each cell nuclei for each worm and measuring the peak fluorescence intensity at the basolateral and apical membranes along that line (Skorobogata et al., 2014). For P6.p cells (L2 and L3), one 20-pixel-wide line was placed across the nucleus, and along the left and right edge of the nucleus, yielding three data points per image. For P6.px and P6.pxx cells, one line was drawn per cell across the nucleus, generating two and four data points per image, respectively. The average basolateral/apical fluorescence intensity ratio across all data points was used for analysis (Fig. 2K).

Mander's correlation coefficients for endogenously tagged LIN-2, LIN-7, LIN-10 and LET-23 EGFR were measured using Zen 2012 software (Zeiss) and quantified by tracing the cells of interest and setting a crosshair threshold that omits background fluorescence and includes cytosolic signal. The same thresholds were used for all images analyzed. The overlap of punctae was determined by identifying punctae using methods described above and checking for overlap in peak fluorescence intensity. The average number of overlapping punctae per worm was used for analysis.

### Analyzing VPC cell fate induction

VPC induction scoring was performed as described previously (Gauthier and Rocheleau, 2017). Each worm was given a VPC induction score between 0 and 6, with scores less than 3 classified as Vul and greater than 3 as Muv. For the vulval induction of worms expressing extrachromosomal arrays, worms expressing GFP in any VPC lineage were scored and compared with siblings on the same plate lacking any detectable extrachromosomal array expression (including the co-injection marker, *pttx-3::gfp*).

### Co-immunoprecipitation

Six nematode growth medium plates saturated with healthy worms kept at 20°C were washed off using sterile M9 buffer and collected in a 15 ml conical tube for each genotype. Harvested worms were washed three times with fresh M9, and two additional times with chilled (4°C) worm protein lysis buffer [50 mM HEPES (pH 7.6), 1 mM EDTA, 1 mM MgCl_2_, 100 mM KCl, 10% glycerol, 0.05% NP-40, cOmplete EDTA-free Protease Inhibitor Cocktail tablet (Sigma-Aldrich), NaF, Na_3_VO_4_ and phenylmethylsulfonyl fluoride]. Worm pellets were resuspended in fresh lysis buffer (up to 2 ml) and freeze/thawed five times in liquid nitrogen, sonicated three times (30% amplitude, 3 s on and 5 s off for a total sonication time of 15 s) until the sample was homogenized, and centrifuged at 12,000 ***g*** for 30 min (4°C). Supernatant was collected as the worm lysate.

SureBeads Protein G Magnetic Beads (Bio-Rad) were washed with 100 μl worm protein lysis buffer and incubated with monoclonal M2 mouse anti-Flag antibody (1:1000, Sigma-Aldrich, F3165) or monoclonal rabbit anti-c-Myc antibody (1:1000, Sigma-Aldrich, PLA0001) in lysis buffer for 1 h at room temperature while rotating on a nutator. Antibody-bound beads were washed with lysis buffer and incubated with 800 µg whole worm lysate overnight at 4°C while rotating. Protein concentration was measured using bovine serum albumin standard assay with Bradford reagent (Bio-Rad). Precipitated proteins were eluted from the beads by boiling in 30 µl 1× SDS sample buffer for 10 min before electrophoresis. A co-immunoprecipitation assay was performed four times for each condition.

SDS-PAGE was performed using TGX Stain-Free FastCast mini gels (Bio-Rad). The protein content on gels was then transferred onto PVDF membranes. Membranes were blocked for 1 h with 5% skimmed milk in 0.1% TBS-T and probed with 1:2000 primary antibody for bait and prey proteins (mouse anti-Flag for LIN-10 or LIN-7, and rabbit anti-c-Myc for LIN-2) diluted in blocking solution and incubated overnight at 4°C while rotating. The next day, membranes were washed with 0.1% TBS-T and incubated with 1:10,000 secondary antibody [rabbit anti-mouse IgG (EMD Millipore, AP160P) or goat anti-rabbit IgG (Sigma-Aldrich, A8275)] diluted in blocking solution for 1 h at room temperature. Membranes were exposed using ECL-Clarity (Bio-Rad) and imaged using a ChemiDoc Imager (Bio-Rad). For bait and prey of a similar size (LIN-10 and LIN-2), the membrane was stripped using a mild stripping buffer (Harlow and Lane, 2006), blocked for 1 h and re-probed. In these cases, prey protein was probed first, then bait protein was probed after stripping.

### Statistical analysis

All statistical analyses were performed using GraphPad Prism 8.0. An unpaired two-tailed Student's *t-*test or one-way ANOVA with Dunnett's test for multiple comparisons were used to compare average means. Fisher's exact test was used to compare vulvaless phenotypes (Vul versus not-Vul), multivulva phenotypes (Muv versus not-Muv), and localization analyses (e.g. punctate versus not punctate).

## Supplementary Material

10.1242/develop.194167_sup1 Supplementary information
