# Supplementary material for: Golgi localization of the LIN-2/7/10 complex points to a role in basolateral secretion of LET-23 EGFR in the Caenorhabditis elegans vulval precursor cells
Source: Development. 2021 Mar 5;148(5):dev194167. doi: 10.1242/dev.194167 (PMC10692275; doi:10.1242/dev.194167)
Supplement: Supplementary information [file develop-148-194167-s1.pdf]

A
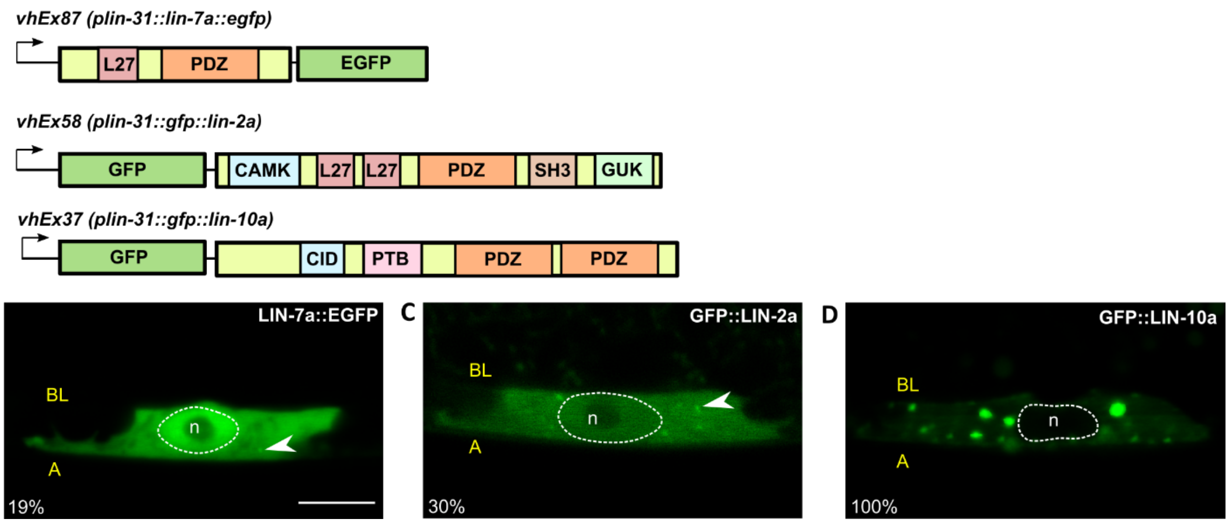

Fig. S1. Localization of LIN-2/7/10 transgenes expressed from extrachromosomal arrays
(A) Schematic of extrachromosomal array transgenes *vhEx87* (*plin-31::lin-7a::egfp*), *vhEx58* (*plin-31::gfp::lin-2a*), and *vhEx37* (*plin-31::gfp::lin-10a*). **(B)** LIN-7a::EGFP is mostly cytosolic and nuclear in VPCs. LIN-7a::EGFP localizes to punctae in 19% of VPCs. **(C)** GFP::LIN-2a localizes diffusely to the cytosol and nucleus in the VPCs. In 30% of VPCs, LIN-2a also localizes to faint cytosolic punctae. **(D)** GFP::LIN-10a localizes to cytoplasmic punctae in all VPCs. Arrowhead: cytosolic punctae in (A, B). Dashed yellow line: outline of the nucleus in (B, C, D). A: Apical. BL: Basolateral. Scalebars: 5 µm.

Development • Supplementary information

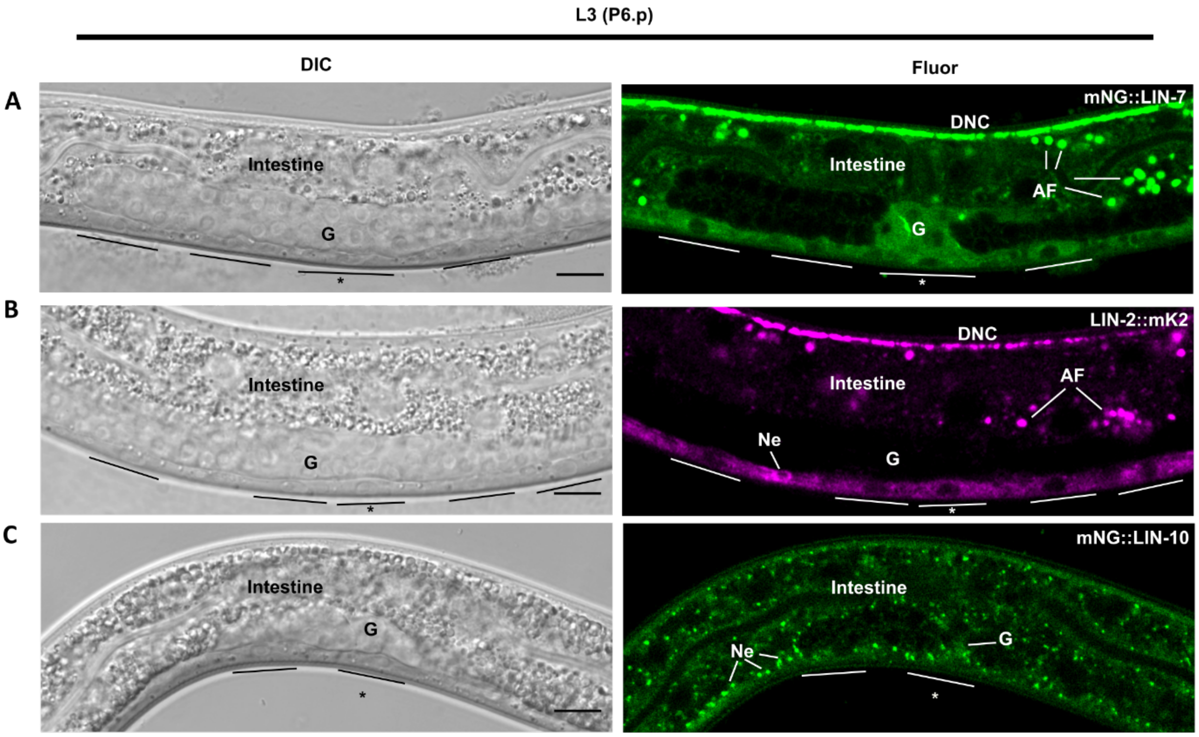

Fig. S2. LIN-2, LIN-7, and LIN-10 are broadly expressed

(A-C) Differential interference contrast (DIC) and corresponding confocal fluorescence images of early L3 larvae (lateral view) expressing endogenously-tagged mNG::LIN-7 (A), LIN-2::mK2 (B) and mNG::LIN-10 (C). VPCs are underlined. Asterisk (*) denotes P6.p cell. G: Gonad. DNC: Dorsal nerve cord. AF: Non-specific autofluorescence in the intestine. Ne: Neuronal cell bodies in the ventral nerve cord. Scalebar: 10 µm.

Development • Supplementary information

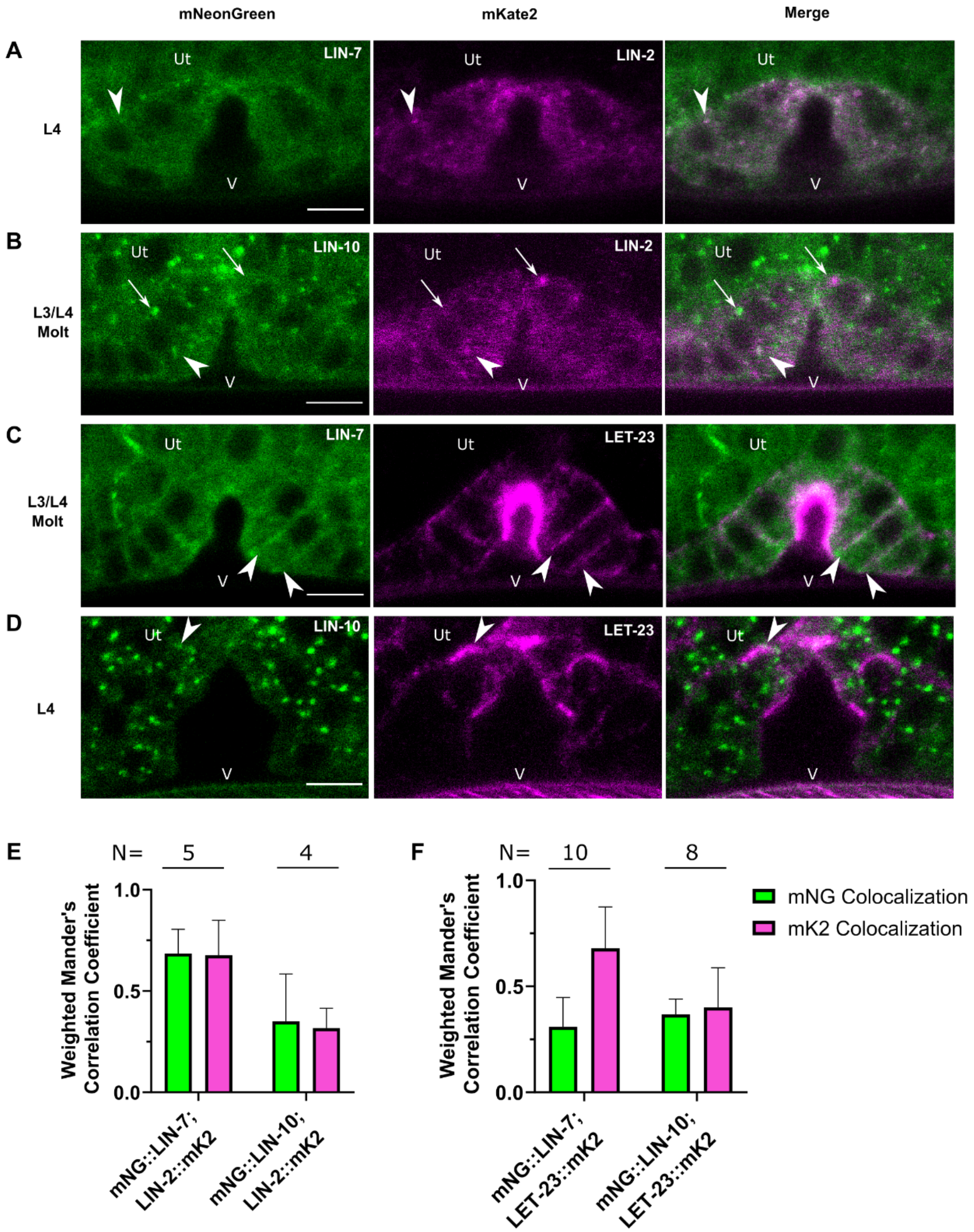

Fig. S3. Colocalization of LIN-2/7/10 and LET-23 EGFR in L4 worms

(A) mNG::LIN-7 and LIN-2::mK2 colocalize in the cytosol and at punctae in L4 worms. **(B)** mNG::LIN-10 and LIN-2::mK2 colocalize at some punctae in L4 worms. **(C)** mNG::LIN-7 and LET-23::mK2 colocalize at basolateral membranes in the developing vulva of L4 worms. **(D)** mNG::LIN-10 and LET-23::mK2 infrequently overlap near cell periphery in L4 worms. **(E)** Weighted Mander's colocalization coefficients for mNG::LIN-7 and mNG::LIN-10 with LIN-2::mK2. **(F)** Weighted Mander's colocalization coefficients for mNG::LIN-7 and mNG::LIN-10 with LET-23::mK2. N: Number of worms analyzed. Scalebars: 5 μm. Arrowhead: colocalizing punctae. Arrow: non-colocalizing punctae. V: Vulval lumen. Ut: L4 uterus. Error bars: SD.

Development • Supplementary information

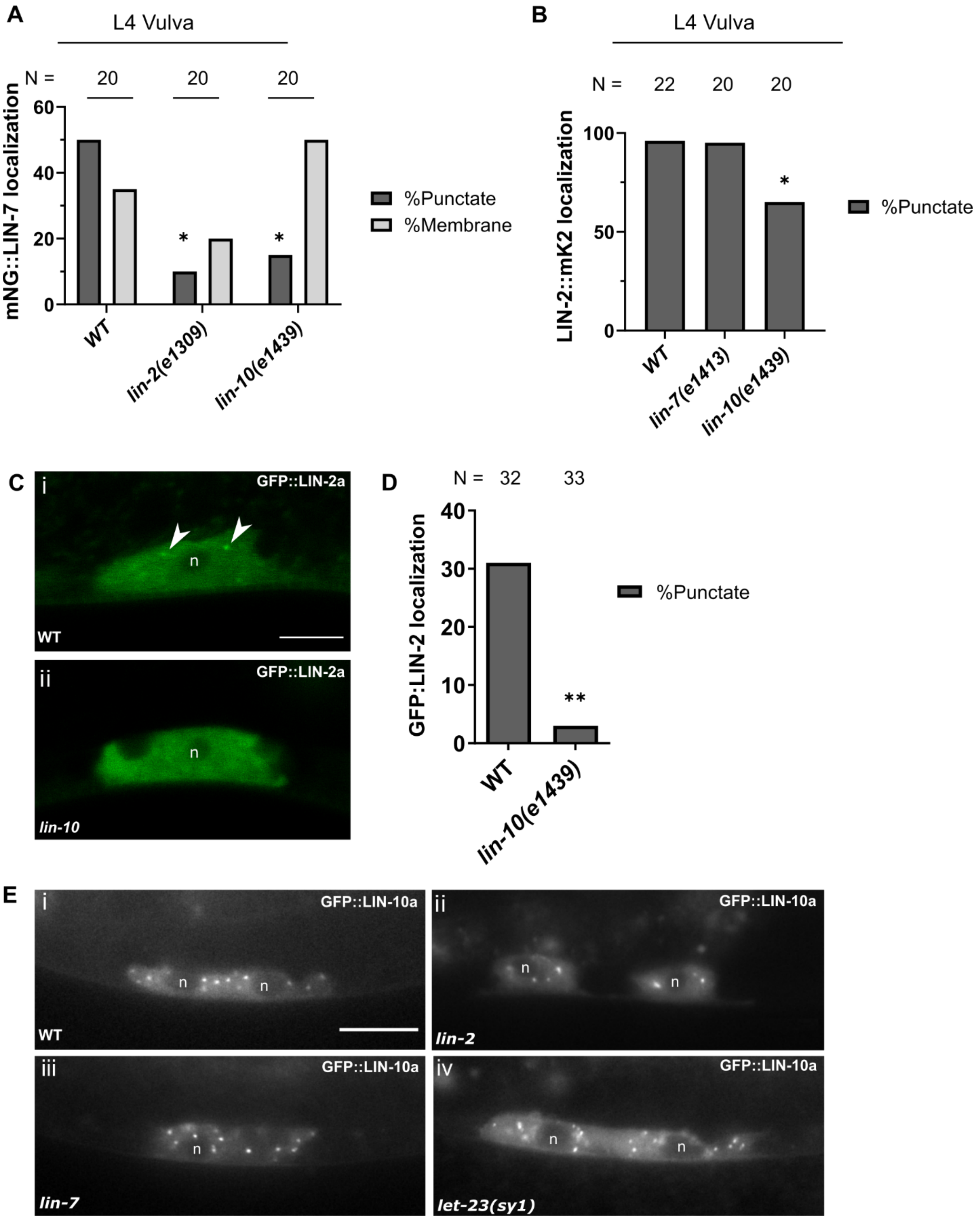

Development • Supplementary information

Fig. S4. Interdependency of LIN-7, LIN-2, and LIN-10 localization

(A) In the developing vulva of L4 worms, mNG::LIN-7 punctate localization is significantly decreased in *lin-2* and *lin-10* mutants. **(B)** In the developing vulva of L4 worms, LIN-2::mK2 punctate localization is significantly decreased in *lin-10* mutants, but not in *lin-7* mutants. **(C, D)** Extrachromosomal GFP::LIN-2a localization in wild type (C.i) and a *lin-10* mutant (C.ii), quantified in (D). Arrowheads: GFP::LIN-2a-positive punctae. n: nucleus. Scalebar: 5 µm. Fisher's Exact Test. **(E)** Extrachromosomal GFP::LIN-10a localizes to punctae in the VPCs of wild type (i) and *lin-2* (ii), *lin-7* (iii), and *let-23(sy1)* (iv) mutants. Scalebar: 10 µm. n: nucleus. N: number of animals scored. *$p<0.05$, **$p<0.01$ Fisher's Exact Test compared to wild type (WT).

Table S1. Analysis of VPC induction in *lin-7(vh51)*, *lin-2(vh52)*, *lin-10(vh50)*, and *let-23(re202)*

Genotype	%Vul	%Muv	Avg # of VPCs induced	n
N2 (wild type)	0%	0%	3.00	36
lin-7(vh51[mNG::3xFlag::LIN-7]) II	0%	0%	3.00	47
lin-2(vh52[LIN-2::mK2::3xMyc]) X	0%	0%	3.00	47
lin-10(vh50[mNG::3xFlag::LIN-10]) I	0%	0%	3.00	45
let-23(re202[LET-23::mK2::3xFlag]) II	5%	7%	3.02	42

One-way ANOVA for VPC induction with Dunnett's test for multiple comparisons. Fisher's exact test for Vul and Muv phenotypes. All conditions compared to N2 (wild type) and no statistical differences were found.

Table S2. Extrachromosomal LIN-7a::EGFP, GFP::LIN-2a, and GFP::LIN-10a rescue their respective mutant phenotypes

	Genotype	%Vul	%Muv	Avg # of VPCs induced	n
1	*lin-7(e1413)*	84%	0%	0.92	25
2	*lin-7(e1413); vhEx87(plin-31::lin-7a::egfp)*	17%****	7%	2.78****	23
3	*lin-2(e1309)*	96%	0%	0.39	23
4	*lin-2(e1309); vhEx58(plin-31::gfp::lin-2a)*	8%****	8%	2.92****	13
5	*lin-10(e1439)*	91%	0%	0.87	22
6	*lin-10(e1439); vhEx37(plin-31::gfp::lin-10a)*	8%****	6%	2.94****	36

Two-tailed Student's *t*-test. Fisher's exact test for Vul and Muv phenotypes compared to the shaded row. ****P<0.0001. Row 2 was compared with row 1. Row 4 was compared with row 3. Row 6 was compared with row 5.

Table S3

Click here to Download Table S3

Development • Supplementary information

Table S4. List of primers used for cloning

Gene	Forward primer (5′ to 3′)	Reverse primer (5′ to 3′)
lin-10 from cDNA	CTATAAGGTACCTCATCTGAAGCAGTAG	GTGACTGAGCTCTCAAATGTATTGTGGTTG
lin-2 from cDNA	TATATAGGTACCAGGGAGCTTGACCCGGAC	GAGCTCTCAGTAGACCCAAGTGACTGGAAG
lin-7 from cDNA	CATATAGTCGACATGGATAACCCGGATGGTC	CATATAACCGGTTCTTCGTGGATTTGTCG
GFP	GAGTCAGCTAGCATGAGTAAAGGAGAAGAAC	GCATGTGGTACCTTGAATTGGTTCCTTTAAAG
EGFP	TATATAACCGGTAGTGCAGGAAGTGCTGCAGGAAGCGGAGAATTTATGGTGAGCAAG	GGTATAGCGGCCGCTTTACTTGTAC
mCherry	CATATAGCTAGCATGGTGAGCAAGGGCGAG	CTATATGGTACCTTGAATTGGTTCCTTTAAAGACTTGTACAGCTCGTCCATG
5′ homology arm for 5′ insertion in *lin-10*	ACGTTGTAAAACGACGGCCAGTCGCCGGCAGTGAACCTAGAACCTAGG	CATGTTGTCCTCCTCTCCCTTGGAGACCATCTTGCAACAGAATTGTTCATAGTC
3′ homology arm for 5′ insertion in *lin-10*	CGTGATTACAAGGATGACGATGACAAGAGAATGTCATCTGAAGCAGTAG	TCACACAGGAAACAGCTATGACCATGTTATGAAGAGGAGAAGACAGG
5′ homology arm for 5′ insertion in *lin-7*	ACGTTGTAAAACGACGGCCAGTCGCCGGCAGTTTTCAACAAATTCG	CATGTTGTCCTCCTCTCCCTTGGAGACCATCTGCAAGATTTGGTTGG
3′ homology arm for 5′ insertion in *lin-7*	CGTGATTACAAGGATGACGATGACAAGAGAATGGATAACCCGGATG	TCACACAGGAAACAGCTATGACCATGTTATCAAATTGCCGATTTGC
5′ homology arm for 3′ insertion in *lin-2*	ACGTTGTAAAACGACGGCCAGTCGCCGGCACCAACATTGTAGGGGTTCATC	CATCGATGCTCCTGAGGCTCCCGATGCTCCGTAGACCCAAGTGACTGGAAG
3′ homology arm for 3′ insertion in *lin-2*	GAGCAGAAGTTGATCAGCGAGGAAGACTTGTGATCTCACACTTTACTAATAC	GGAAACAGCTATGACCATGTTATCGATTTCCAAACAGTTACTCTCTTCTGTC
Verification of 5′ insertion in *lin-10*	CAGTTTCCACCTATCATAATTGGTCC	CTGATATTGAGCTGGAGAGAATACCATC
Verification of 5′ insertion in *lin-7*	GTGATGCAATGCAACTC	CGTGTTGTTAAGCGTATC
Verification of 3′ insertion in *lin-2*	GTAAAAACTACAGGGTTGATCC	GAATTTAATTTCTTTTTGTCGTC

Development • Supplementary information
