# Supplementary material for: Golgi localization of the LIN-2/7/10 complex points to a role in basolateral secretion of LET-23 EGFR in the Caenorhabditis elegans vulval precursor cells
Source: Development. 2021 Mar 5;148(5):dev194167. doi: 10.1242/dev.194167 (PMC10692275; doi:10.1242/dev.194167)
Supplement: Supplementary file 1 [file develop-148-194167_review_history.pdf]

Golgi localization of the LIN-2/7/10 complex points to a role in basolateral secretion of LET-23 EGFR in the *Caenorhabditis elegans* vulval precursor cells

Kimberley D Gauthier and Christian E Rocheleau
DOI: 10.1242/dev.194167

Editor: Swathi Arur
Review timeline
Original submission: 18 June 2020
Editorial decision: 5 August 2020
First revision received: 16 December 2020
Editorial decision: 14 January 2021
Second revision received: 22 January 2021
Accepted: 25 January 2021

Original submission

First decision letter

MS ID#: DEVELOP/2020/194167

MS TITLE: Dynamic expression and localization of the LIN-2/7/10 protein scaffolding complex during *C. elegans* vulval development

AUTHORS: Kimberley D Gauthier and Christian E Rocheleau

I have now received reviews of your manuscript from 3 experts. The reviewers' comments are appended below, or you can access them online: please go to BenchPress and click on the 'Manuscripts with Decisions' queue in the Author Area.

As you will see, all 3 reviewers express interest in your study but have some concerns. They offer numerous excellent suggestions to address those concerns and improve your study and manuscript. Reviewer 1 requests additional experiments, including analysis of larvae across critical stages of vulval cell induction and differentiation, time-lapse imaging of LET-23 in wild type and mutants, and identification of the compartment(s) involved in LIN-2/7/10 trafficking. Reviewer 2 (in confidential comments to me) and I both agree with Reviewer 1's requests. We realize that accessing the lab may be impossible or slow during the pandemic, but we think that strengthening your paper along the experimental lines suggested is critical for your paper to meet the criteria for publication in Development. Reviewer 2 further recommends revising the text to better move your study beyond descriptive and to make clearer the novelty of the conclusions and the significance of the advance. Such revisions would also likely address Reviewer 3's concern that the final conclusions are to some extent suggestions/speculations.

I invite you to consider the reviewers' suggestions and submit a revised manuscript that addresses their concerns. Your revised manuscript would be re-reviewed, and acceptance would depend on your satisfactorily addressing the reviewers' concerns. Please note that Development normally permits only one round of â€˜major revision' and that because of the pandemic we are happy to extend revision time frames as necessary.

© 2021. Published by The Company of Biologists under the terms of the Creative Commons Attribution License (https://creativecommons.org/licenses/by/4.0/).

In your revised manuscript, please clearly HIGHLIGHT all changes made in the revised version. You should avoid using 'Tracked Changes' in Word files as these are lost in PDF conversion. I also request a point-by-point response detailing how you have dealt with the points raised by the reviewers in the 'Response to Reviewers' box. If you do not agree with any of the reviewersâ€™ criticisms or suggestions, please explain why.

Reviewer 1

Advance summary and potential significance to field

LIN-2, -7, -10 are evolutionarily conserved, interacting proteins previously shown to promote basolateral localization of C. elegans LET-23/EGFR. In the 1998 Kaech et al publication providing evidence for this role, the question was left open as to whether LIN-2/7/10 influenced basolateral trafficking of LET-23 or acted at the basolateral plasma membrane to anchor LET-23 there or to prevent its endocytosis. In the 20+ years since then, the issue still has not been resolved. LIN-7 was reported to co-localize with LET-23 at basolateral membranes and junctions (based on overexpressed transgene reporters), but the location of the rest of the complex in vulva cells remained unclear. Here, Gauthier and Rocheleau revisit the question using endogenously expressed LET-23, LIN-2, LIN-7, and LIN-10 fusions (tagged using CRISPR genome editing). They visualize the localization of each fusion over developmental time, test for colocalization and for mis-localization in mutant backgrounds, and use the fusions for in vivo co-IP expts. The results clearly show that LIN-2, -7 and -10 are often found separately and co-localize in endosomes rather than at basolateral membranes, and also reveal previously unknown LIN-2/LIN-7 subcomplexes and a LIN-10 --> LIN-2 --> LIN-7 regulatory hierarchy for endosome recruitment. The results suggest a role for the complex in initial basolateral trafficking or recycling of LET-23 rather than in plasma membrane retention (though LIN-7 could play roles in both processes).

This manuscript is well written and the reagents and data included here are a very important step forward for understanding the role of LIN-2/7/10. Evidence for a role in basolateral trafficking is still fairly circumstantial, but the authors are careful in their language and claims (perhaps too careful in the title, which is quite vague). I have several questions and concerns as summarized below.

Comments for the author

1. A lot of imaging here is done at stages long after LET-23-dependent vulva fate induction. The authors should give more rationale for why looking at these later stages is relevant. Were any L2 or earlier L3 stages examined, and why isn't there more focus on those stages?

2. There seems to be a disconnect between the dogmatic (but logical) idea that the amount of LET-23 at the basolateral surface really matters for signaling, vs. the large differences in the basal::apical ratios seen with different functional LET-23 reagents. e.g. in Figure 5, LET-23::mKate2 is reasonably functional, yet barely observable at basolateral surfaces, looking much more like zhIs35 (transgenic LET-23::GFP) in a lin-2 mutant. This makes me wonder how much of the truly functional pool of protein we are seeing with any of these reagents. Can the authors comment on this? (could it be related to looking at too late of a stage? or is the slower maturation time for mKate2 masking the signal? or is the amount of LET-23 actually needed at the PM just very low?)

3. It is disappointing that the trafficking compartments where these proteins are found are not identified more precisely. Only one marker, VPS-52, is used to support the idea that the observed puncta correspond to Golgi ministacks or recycling endosomes. Additional markers could distinguish among these possibilities. Another thing that would benefit this field is time-lapse imaging of LET-23 in WT vs. mutants, to test whether initial targeting, endocytosis or recycling is affected. I understand that COVID may preclude such experiments any time soon.

4. In the Discussion, can the authors mention how their model explains the observation that LET-23 accumulates at apical membranes in the lin-2/7/10 mutants? And also how it explains the

© 2021. Published by The Company of Biologists under the terms of the Creative Commons Attribution License (https://creativecommons.org/licenses/by/4.0/).

observations of Stetak et al (2006), who found that in lin-2 mutants, LET-23 accumulated either in puncta or at the apical membrane, depending on which LIN-2 domain was mutated?

5. lines 418-421: "slightly but not significantly" and "appeared to be LIN-10 dependent". Isn't the point of the statistics to say that the differences observed are not more than one would expect by chance? in which case why mention them as though they are meaningful?

Additional minor comments on figures:
6. Most of the early figures (Figs 1,2) are too zoomed out for me to easily see the localization patterns that are being discussed. I really need to see images like those of Figure S1 or Figure 8 right off the bat. (and Figure 8 is cited out of order, on lines 245 and 255 early in Results).

7. Figure 1 vs. FigureS1: Comparison of N- and C-terminally tagged LIN-7 is difficult since the former is endogenous and the latter over-expressed. However, the difference in their localization patterns is striking. Have Westerns been done with the C-term tagged version to check if LIN-7 is proteolytically cleaved?

8. Figure 2I,J: At P6.p stage, the LET-23 basolateral signal appears much weaker than the apical signal visually, yet the graph is normalized to show their ratio as 1; this is very non-intuitive and confusing.

9. Figure 3. The title doesn't make sense given the obvious expression outside of vulva cells. Rephrase.

10. Figure 4C,D,E,F,G. It would be helpful if labels re stage were added here. G is confusing and stats are not indicated.

11. Figure 5. As mentioned above, there seems to be a disconnect between the idea that the amount of LET-23 at the basolateral surface really matters for signaling and the localization shown here for LET-23::mKate2. Also, too much of the imaging here is done at late stages, when it's hard to say what any co-localization means. Overall I found this figure very confusing!

12. Figure 5C,D,E,F,G. It would be helpful if labels re stage were added here.

13. Figure 8: Why are data in panels B/C vs. E/F shown in different bar graph formats? It makes it harder to compare the results.

14. Figure 9: mislabelled as Figure 1 in legend. Mander's coefficients should be calculated here as in the other Figures.

15. Figure 9 C,D,E: In prior figures, arrowheads indicated puncta in the relevant vulva cells and arrows indicated puncta in other cells. Now here the symbols are reversed.

16. Table S3 legend mentions various statistical tests but no results of such tests are indicated.

Reviewer 2

Advance summary and potential significance to field

Background relevant to the work:
Developmental patterning of VPC fates is a classic developmental system. Mutations in lin-2, -7, and -10 were identified by virtue of causing a vulvaless phenotype. The LIN-2/7/10 complex is required for localization of EGFR/LET-23 to the basolateral surface of VPCs, as is the C-terminal 6 residues of LET-23/EGFR. Vulvaless mutants have aberrant increased apical localization of LET-23 and no visible basolateral localization. This model was established in a series of papers from Stuart KimÂ’s lab in the 90s.

Advance: meticulous analysis of the endogenous proteins of the LIN-2/7/10 complex with let-23, showing co-localization and co-IPs. This is strong work, yet I am ambivalent about whether there is

© 2021. Published by The Company of Biologists under the terms of the Creative Commons Attribution License (https://creativecommons.org/licenses/by/4.0/).

a significant advance vs. incremental, albeit beautiful, work. Some of the reservation may be based on the failure of the authors to clearly delineate what was novel about their conclusions

Comments for the author

Summary of the work:
Here the authors use modern genome editing technology to tag endogenous LIN-2, LIN-7 and LIN-10 proteins. With these reagents they analyze the localization and co-localization of LIN-2/7/10 proteins and LET-23/EGFR, as well as binding within the complex by co-immunoprecipitation. Importantly, by analyzing endogenous proteins, the authors establish the most rigorous look at the functions of this complex. The analysis is careful and comprehensive, and represents an important validation long-standing models. Enthusiasm is dampened somewhat by the descriptive nature of the work and the absence of novel insights.

Overall assessment:
The study is careful and rigorous and this reviewer does not have major scientific concerns. Rather, I would focus on the writing narrative and whether the authors are missing major conclusions. (The answer from me: I don't know). This question arises from the Summary Statement: "LIN-10 recruits LIN-2 and LIN-7 to Golgi or recycling endosomes, consistent with targeting rather than tethering the epidermal growth factor receptor to the basolateral membrane in C. elegans." This point is also emphasized in the Introduction, but is not further discussed. Is this an important model? It is not clear to me what the authors are thinking about this issue and why it is important. Beyond that, I enjoyed the work but wondered whether it was too incremental and descriptive for Development. For example, the title sounds descriptive, e.g. "Dynamic expression and localization." Is the work that novel? Re-working of the writing to emphasize the novel advances would increase enthusiasm. And nonetheless, I find the study an important advance because of its rigor. This is the future of how such analyses need to be completed, and it is worth revisiting this question.

Major concerns:
No major experimental concerns.

Other concerns and suggestions:
1. Describe validation of CRISPR tags. Sequencing of regions subjected to HDR. In methods, call out Fig 7 for size validation of CRISPR knockins, note that size is expected (if so).
2. Fig 1B seems like a lost opportunity: why not show domains in the proteins for members of the complex so readers can remember the functions amongst the alphabet soup? Can also mention mammalian homologue here, so we have a cheat sheet 3. Line 106: burying the lede: important point in the middle of a paragraph 4. Line 107: "apical junctions": you want to be precise. These are adherens junctions, right?
5. Last two paragraphs of Intro: the narrative lacks punch. How can you rearrange this section to generate enthusiasm for the work? And shouldn't the discussion circle back to reiterate these points, and the central point of the Summary Statement.
6. More narrative concerns from the Intro: last sentence, these conflicting models have not been delineated. Is this important? How about setting this up as an important question and then resolve it? Identify the gap, then fill it.
7. Line 916: Figure 10, not 1 8. Shouldn't we see quantification between VPC lineages after induction? After all, these cells assume different fates, so a comparison between presumptive primary, presumptive secondary and presumptive tertiary seems like valuable data to have out there. There is, after all, some dynamic change in expression in this system during differentiation.The good news is that the authors likely have these data in existing images, so no need to do further experiments.
9. Also, is localization altered in different VPC lineages?
10. re202 tagging position sounds very similar to that described for zhIs035 (Haag), which raises the question of whether zhIs35 also has compromised function. Would we know? This should be noted.

© 2021. Published by The Company of Biologists under the terms of the Creative Commons Attribution License (https://creativecommons.org/licenses/by/4.0/).

Reviewer 3

Advance summary and potential significance to field

This manuscript presents the results of a detailed analysis of the expression pattern of the components of the LIN-2/LIN-7/LIN-10 complex during development of the C. elegans vulva. This complex has been previously shown to recruit/stabilise the EGF receptor orthologue, LET-23, on the basolateral membrane of the vulva precursor cells (VPCs). This is important to properly specify the vulva fate. But how the complex performs this function and how the components of the complex are localised themselves remained elusive so far.
To address this question, the authors performed a very detailed analysis of the expression pattern of the components of the complex, both in wild-type and in mutant animals. Except for LIN-10, all others show dynamic expression during development. From the analysis of localisation of each of the members in mutants of the respective other genes the authors conclude that LIN-2 and LIN-7 depend on LIN-10 for their proper localisation on cytoplasmic punctae. LET-23 co-localises with LIN-7 on the basolateral plasma membrane and with the LIN-2/LIN-7/LIN-10 complex on intracellular punctae, which are likely to correspond to Golgi stacks or recycling endosomes. LET-23 can also be found on these punctae.

Given that the members of this complex and their role in the localisation of LET-23 have been published more than 20 years ago, it is surprising how little we still know about their specific in-vivo role during vulva development. Therefore, the data presented here shed more light on the how the complex may be built and how it may control the targeting of the receptor to the basolateral membrane. Given that the data are mostly obtained from localisation/expression of GFP-tagged proteins, the final conclusions are to some extend suggestions/speculations. But these can now be tested with more sophisticated methods.

Comments for the author

I have a few points that were not addressed:
a. The analysis is nearly exclusively based on fluorescently tagged proteins. Although the authors (or others) showed that these can rescue the respective mutant phenotypes, the localisation may still not reflect the “normal” pattern and dynamic, since immuno-fluorescent images only take snapshots.
b. In addition, some of these proteins are subjected to posttranslational modifications as shown in other systems, and the genes may encode more than one isoform.
c. Furthermore, it has been shown previously that other molecules (ERM-1, EPS-8) are involved in some aspects of receptor trafficking/stabilisation. How does this relate to the LIN-2/LIN-7/LIN-10 complex?
I suggest that the authors at least discuss these aspects.

First revision

Author response to reviewers' comments

Reviewer 1 Advance Summary and Potential Significance to Field:
LIN-2, -7, -10 are evolutionarily conserved, interacting proteins previously shown to promote basolateral localization of C. elegans LET-23/EGFR. In the 1998 Kaech et al publication providing evidence for this role, the question was left open as to whether LIN-2/7/10 influenced basolateral trafficking of LET-23 or acted at the basolateral plasma membrane to anchor LET-23 there or to prevent its endocytosis. In the 20+ years since then, the issue still has not been resolved. LIN-7 was reported to co-localize with LET-23 at basolateral membranes and junctions (based on overexpressed transgene reporters), but the location of the rest of the complex in vulva cells remained unclear. Here, Gauthier and Rocheleau revisit the question using endogenously expressed LET-23, LIN-2, LIN-7, and LIN-10 fusions (tagged using CRISPR genome editing). They visualize the localization of each fusion over developmental time, test for colocalization and for mis-localization

© 2021. Published by The Company of Biologists under the terms of the Creative Commons Attribution License (https://creativecommons.org/licenses/by/4.0/).

in mutant backgrounds, and use the fusions for in vivo co-IP expts. The results clearly show that LIN-2, -7 and -10 are often found separately and co-localize in endosomes rather than at basolateral membranes, and also reveal previously unknown LIN-2/LIN-7 subcomplexes and a LIN-10 --> LIN-2 --> LIN-7 regulatory hierarchy for endosome recruitment. The results suggest a role for the complex in initial basolateral trafficking or recycling of LET-23 rather than in plasma membrane retention (though LIN-7 could play roles in both processes).

This manuscript is well written and the reagents and data included here are a very important step forward for understanding the role of LIN-2/7/10. Evidence for a role in basolateral trafficking is still fairly circumstantial, but the authors are careful in their language and claims (perhaps too careful in the title, which is quite vague). I have several questions and concerns as summarized below.

Reviewer 1 Comments for the Author:

1. A lot of imaging here is done at stages long after LET-23-dependent vulva fate induction. The authors should give more rationale for why looking at these later stages is relevant. Were any L2 or earlier L3 stages examined, and why isn't there more focus on those stages?

The absence of analysis of LIN-2/7/10 localization in the VPCs of early L2 animals was an oversight. We imaged early L2 animals expressing LIN-2::mK2, mNG::LIN-7, mNG::LIN-10 and LET-23::GFP and included these data in our analysis of expression across the different stages of vulva development in Figure 2. We also updated Figure 1 to include early L2 images. We did not detect any significant differences in localization between VPCs in L2 and L3 stages. We looked at later stages of vulva development because the proteins continue to be expressed and the expression of many regulators of signaling are themselves regulated by signaling. We have better articulated this in the manuscript. We have also focused the paper more on the earlier stages of vulva development and moved much of the analysis of later stages to supplemental figures since it only supports our findings in the VPCs.

2. There seems to be a disconnect between the dogmatic (but logical) idea that the amount of LET-23 at the basolateral surface really matters for signaling, vs. the large differences in the basal::apical ratios seen with different functional LET-23 reagents. e.g. in Figure 5, LET-23::mKate2 is reasonably functional, yet barely observable at basolateral surfaces, looking much more like zhIs35 (transgenic LET-23::GFP) in a lin-2 mutant. This makes me wonder how much of the truly functional pool of protein we are seeing with any of these reagents. Can the authors comment on this? (could it be related to looking at too late of a stage? or is the slower maturation time for mKate2 masking the signal? **or is the amount of LET-23 actually needed at the PM just very low?)**

We believe that the amount of LET-23 required on the basolateral membrane is very low. We added a section on LET-23 EGFR localization and the regulation of its apical and basolateral localization in the Discussion and discuss the data that supports our view.

3. It is disappointing that the trafficking compartments where these proteins are found are not identified more precisely. Only one marker, VPS-52, is used to support the idea that the observed puncta correspond to Golgi ministacks or recycling endosomes. Additional markers could distinguish among these possibilities. Another thing that would benefit this field is time-lapse imaging of LET-23 in WT vs. mutants, to test whether initial targeting, endocytosis or recycling is affected. <u>I understand that COVID may preclude such experiments any time soon.</u>

We agree that the ambiguity of the trafficking compartments weaken the study. We have now looked at Golgi (AMAN-2) and ER (SP12 or SPCS-1) markers with both LIN-10 and LIN-7 and found that LIN-10 and LIN-7 do overlap with the Golgi, cementing the idea that the Golgi is the site of complex formation. These data are included in Fig. 9.

We agree that there remains a big gap in our understanding of the trafficking routes by which LET-23 reaches the apical and basolateral membrane. It has been proposed that it appears on the apical membrane first and then could translocated to the basolateral membrane. However, since there is more LET-23 present on the apical membrane, we don’t know if it goes there first, or just becomes visible first because it is more abundant. We attempted some time-lapse imaging of LET-23::GFP

© 2021. Published by The Company of Biologists under the terms of the Creative Commons Attribution License (https://creativecommons.org/licenses/by/4.0/).

but did not see anything enlightening, however we were hampered by too much movement of the animals. Further troubleshooting using microfluidics would help. We also think generating a new CRISPR strain with a brighter tag, and testing alternative sites for fluorophore tagging to resolve the mild disruption of LET-23 function observed in the LET-23::mK2 line, would be the best way forward to addressing these questions. We think the ideas suggested by the reviewer, coupled with photobleaching experiments, and structure/function analysis of the LET-23 cytoplasmic tail with regards to localization would resolve the trafficking routes of LET-23 in the future.

4. In the Discussion, can the authors mention how their model explains the observation that LET-23 accumulates at apical membranes in the lin-2/7/10 mutants? And also how it explains the observations of Stetak et al (2006), who found that in lin-2 mutants, LET-23 accumulated either in puncta or at the apical membrane, depending on which LIN-2 domain was mutated?

In the discussion we added a section on LET-23 EGFR localization and discuss our thoughts that the apical membrane localization appears to be a default localization in the VPCs and that the LIN-2/7/10 complex is required to potentially redirect LET-23 to the basolateral membrane.

LIN-2 appears to have dual roles mediated by its two L27 domains. The second L27 domain mediates its interaction with LIN-7, but the first interacts with EPS-8 to regulate endosomal trafficking of LET-23. We discuss the findings of Stetak et al 2006 in the context of our data.

5. lines 418-421: "slightly but not significantly" and "appeared to be LIN-10 dependent". Isn't the point of the statistics to say that the differences observed are not more than one would expect by chance? in which case why mention them as though they are meaningful?

Agreed! We removed “slightly”.

Additional minor comments on figures:

6. Most of the early figures (Figs 1,2) are too zoomed out for me to easily see the localization patterns that are being discussed. I really need to see images like those of Figure S1 or Figure 8 right off the bat. (and Figure 8 is cited out of order, on lines 245 and 255 early in Results).

We redesigned figures 1 and 2 to improve clarity. In Figure 1: we moved the original set of zoomed out images into a new Supplementary Figure 2. We kept these images as reference for some expression patterns of LIN-2/7/10 in other tissues. In the new Figure 1, we added zoomed in images on the P6.p cells at the early L2 stage as well 2-cell P6.px cells (because this is where membrane-localized LIN-7 is most apparent). In Figure 2, we reorganized the images and graphs to increase the size of each image, making the localization patterns easier to see.

7. Figure 1 vs. FigureS1: Comparison of N- and C-terminally tagged LIN-7 is difficult since the former is endogenous and the latter over-expressed. However, the difference in their localization patterns is striking. Have Westerns been done with the C-term tagged version to check if LIN-7 is proteolytically cleaved?

Thank you for pointing this out. We noticed that we missed a frameshift in our construct that puts the C-terminally tagged GFP out of frame from LIN-7 and results in an extension of the LIN-7 protein that apparently does not disrupt its function. We were thrown off by the fact that GFP apparently does express from its own start codon. Therefore, it seems GFP and LIN-7 were being co-expressed, but not as a fusion protein, which is why we were able to get rescue of *lin-7* mutants and still see some GFP signal. This means that the localization we saw for LIN-7::EGFP was not accurate. We remade this construct and repeated our rescue and localization experiments and found that LIN-7::EGFP fusion protein resembles that of the mNG::LIN-7 being highly cytosolic and does localize to cytosolic puncta (though very infrequently). However, we did not see basolateral membrane localization, possibly due to interference by the C-terminal tag, yet it strongly rescues the *lin-7* mutant.

8. Figure 2I,J: At P6.p stage, the LET-23 basolateral signal appears much weaker than the apical signal visually, yet the graph is normalized to show their ratio as 1; this is very non-intuitive and

© 2021. Published by The Company of Biologists under the terms of the Creative Commons Attribution License (https://creativecommons.org/licenses/by/4.0/).

confusing.

Originally, the ratios at P6.px and P6.pxx were normalized to the ratio found at the P6.p stage. We did that so that we could have the three lines on the graph start from the same point, but we see that it is confusing and non-intuitive. We simplified the figure to just show the basolateral/apical intensity ratio percentages at each developmental stage. We also added the early L2 P6.p stage for this analysis.

9. Figure 3. The title doesn't make sense given the obvious expression outside of vulva cells. Rephrase.

We changed the title to better reflect the result that LIN-2 and LIN-7 expression is reduced in the descendants of the uninduced VPCs. Although LIN-2 and LIN-7 are broadly expressed, their expression is qualitatively lower in some tissues than others. We have not analyzed Hyp7 in depth, but it appears to have lower expression levels as compared to the vulva and neurons.

10. Figure 4C,D,E,F,G. It would be helpful if labels re stage were added here. G is confusing and stats are not indicated.

We simplified this image to show everything at the same stage: early L3 P6.p cells (instead of P6.px cells as in the original). Images and graphs at L4 were moved to a new Supplementary Figure 3. Labels were added to Fig 4C,D (formerly 4E,F). Fig 4E (formerly 4G) was simplified as well: originally, we were trying to demonstrate that there were fewer worms that had punctate LIN-7 in a *lin-2* mutant, but the few puncta that were present overlapped with LIN-10. However, the representation was confusing, and the graph was a bit squished. Instead, we simply show that the % overlap is unchanged in a *lin-2* mutant. We added sample sizes (total number of puncta observed, with number of animals in brackets). Because we already show that punctate LIN-7 decreases in a *lin-2* mutant in Fig 8, it was redundant to show it in Fig 4 as well.

11. Figure 5. As mentioned above, there seems to be a disconnect between the idea that the amount of LET-23 at the basolateral surface really matters for signaling and the localization shown here for LET-23::mKate2. Also, too much of the imaging here is done at late stages, when it's hard to say what any co-localization means. Overall I found this figure very confusing!

We moved the L4 data from this figure into the new Supplementary Figure 3 so that the main figure can be focused on more relevant stages of VPC induction. Basolateral localization of LIN-7 is rare at the 1-cell P6.p stage, and is almost undetectable for LET-23::mKate2. Due to these limitations (particularly those posed by the LET-23 CRISPR line), we show overlap at P6.px.

12. Figure 5C,D,E,F,G. It would be helpful if labels re stage were added here.

As mentioned above, we moved later stage data to supplemental figure 3, and labels were added.

13. Figure 8: Why are data in panels B/C vs. E/F shown in different bar graph formats? It makes it harder to compare the results.
The punctate and membrane localization patterns for lin7 are not mutually exclusive; this is why these bars were represented separately. However, to simplify the data, we removed the “cytosolic” bar (as this is simply the absence of membrane and puncta). This way, the localization data for lin7 and lin2 are represented in more similarly. Also, for this figure, we moved the graphs for L4 vulva into a new Supplementary Figure 4, and we added the localization of mNG::LIN-10 which was previously part of figure 9.

14. Figure 9: mislabelled as Figure 1 in legend. Mander's coefficients should be calculated here as in the other Figures.

Label has been corrected. We added Mander’s coefficients for LIN-10 colocalization with subcellular markers.

For LIN-7, because of the high cytosolic expression, we found that the % overlap of LIN-7 puncta with the subcellular markers to be the best way to understand colocalization of LIN-7 instead of

© 2021. Published by The Company of Biologists under the terms of the Creative Commons Attribution License (https://creativecommons.org/licenses/by/4.0/).

using Mander's coefficients, which was too sensitive to the cytosolic fraction. To be consistent, we also looked at % overlap of LIN-10 puncta, which showed similar patterns as the Mander's coefficients.

15. Figure 9 C,D,E: In prior figures, arrowheads indicated puncta in the relevant vulva cells and arrows indicated puncta in other cells. Now here the symbols are reversed.

We switched the symbols in this figure to be more consistent with previous figures.

16. Table S3 legend mentions various statistical tests but no results of such tests are indicated.

We added to the legend that there were no statistical differences.

Reviewer 2 Advance Summary and Potential Significance to Field:
Background relevant to the work:
Developmental patterning of VPC fates is a classic developmental system. Mutations in lin-2, -7, and -10 were identified by virtue of causing a vulvaless phenotype. The LIN-2/7/10 complex is required for localization of EGFR/LET-23 to the basolateral surface of VPCs, as is the C-terminal 6 residues of LET-23/EGFR. Vulvaless mutants have aberrant increased apical localization of LET-23 and no visible basolateral localization. This model was established in a series of papers from Stuart Kim's lab in the 90s.

Advance: meticulous analysis of the endogenous proteins of the LIN-2/7/10 complex with let-23, showing co-localization and co-IPs. This is strong work, yet I am ambivalent about whether there is a significant advance vs. incremental, albeit beautiful, work. Some of the reservation may be based on the failure of the authors to clearly delineate what was novel about their conclusions

Reviewer 2 Comments for the Author:
Summary of the work:
Here the authors use modern genome editing technology to tag endogenous LIN-2, LIN-7 and LIN-10 proteins. With these reagents they analyze the localization and co-localization of LIN-2/7/10 proteins and LET-23/EGFR, as well as binding within the complex by co-immunoprecipitation. Importantly, by analyzing endogenous proteins, the authors establish the most rigorous look at the functions of this complex. The analysis is careful and comprehensive, and represents an important validation long-standing models. Enthusiasm is dampened somewhat by the descriptive nature of the work and the absence of novel insights.

Overall assessment:
The study is careful and rigorous and this reviewer does not have major scientific concerns. Rather, I would focus on the writing narrative and whether the authors are missing major conclusions. (The answer from me: I don't know). This question arises from the Summary Statement: "LIN-10 recruits LIN-2 and LIN-7 to Golgi or recycling endosomes, consistent with targeting rather than tethering the epidermal growth factor receptor to the basolateral membrane in C. elegans." This point is also emphasized in the Introduction, but is not further discussed. Is this an important model? It is not clear to me what the authors are thinking about this issue and why it is important. Beyond that, I enjoyed the work but wondered whether it was too incremental and descriptive for Development. For example, the title sounds descriptive, e.g. "Dynamic expression and localization." Is the work that novel? Re-working of the writing to emphasize the novel advances would increase enthusiasm. And nonetheless, I find the study an important advance because of its rigor. This is the future of how such analyses need to be completed, and it is worth revisiting this question.

Major concerns:
No major experimental concerns.

Other concerns and suggestions:
1.Describe validation of CRISPR tags. Sequencing of regions subjected to HDR. In methods, call out Fig 7 for size validation of CRISPR knockins, note that size is expected (if so).

This was added to the Materials and Methods.

© 2021. Published by The Company of Biologists under the terms of the Creative Commons Attribution License (https://creativecommons.org/licenses/by/4.0/).

2.Fig 1B seems like a lost opportunity: why not show domains in the proteins for members of the complex so readers can remember the functions amongst the alphabet soup? Can also mention mammalian homologue here, so we have a cheat sheet

Great idea! We added protein domains to CRISPR alleles illustrated in Fig 1C,E,G (formerly Fig 1B).

3.Line 106: burying the lede: important point in the middle of a paragraph

Great point. We reorganized the introduction and this lead is now at the front of the paragraph.

4.Line 107: “apical junctions”: you want to be precise. These are adherens junctions, right?

Technically, the *C. elegans* apical junction refers to both the adherens junction and the adjacent AJM-1/DLG-1 complex. Since Simske et al 1996 found partial colocalization between LIN-7 and AJM-1, we more precisely refer to it as the apical junction marker (AJM-1) in the text.

5.Last two paragraphs of Intro: the narrative lacks punch. How can you rearrange this section to generate enthusiasm for the work? And shouldn’t the discussion circle back to reiterate these points, and the central point of the Summary Statement.

We agree that we failed to emphasize the novelty and significance of our work. We reconstructed the introduction to build a better narrative and rewrote large parts of the discussion to better highlight the importance of our findings.

6.More narrative concerns from the Intro: last sentence, these conflicting models have not been delineated. Is this important? How about setting this up as an important question and then resolve it? Identify the gap, then fill it.

See reply for comment 5 and we also added an illustration in Fig 1B to further highlight the central problem we are trying to address.

7.Line 916: Figure 10, not 1

Corrected.

8.Shouldn’t we see quantification between VPC lineages after induction? After all, these cells assume different fates, so a comparison between presumptive primary, presumptive secondary and presumptive tertiary seems like valuable data to have out there. There is, after all, some dynamic change in expression in this system during differentiation.The good news is that the authors likely have these data in existing images, so no need to do further experiments.

Qualitatively did not see any differences between the primary and secondary lineages, so we did not follow up on this. Unfortunately, we don’t have enough data in our current image set to properly quantify if there was indeed a small difference or not.

9.Also, is localization altered in different VPC lineages?

As above, we did not look in depth at the secondary lineages, but this could be pursued in the future.

10.re202 tagging position sounds very similar to that described for zhIs035 (Haag), which raises the question of whether zhIs35 also has compromised function. Would we know? This should be noted.

This is a good point. I don’t think we know whether zhIs035 is similarly compromised. It rescues let-23(sy1), but a better test would be rescue of a signaling deficient allele such as let-23(sy97). It is a low copy number insertion; extra copies could potentially compensate for a small alteration in the function of LET-23 by the GFP tag.

© 2021. Published by The Company of Biologists under the terms of the Creative Commons Attribution License (https://creativecommons.org/licenses/by/4.0/).

Reviewer 3 Advance Summary and Potential Significance to Field:
This manuscript presents the results of a detailed analysis of the expression pattern of the components of the LIN-2/LIN-7/LIN-10 complex during development of the C. elegans vulva. This complex has been previously shown to recruit/stabilise the EGF receptor orthologue, LET-23, on the basolateral membrane of the vulva precursor cells (VPCs). This is important to properly specify the vulva fate. But how the complex performs this function and how the components of the complex are localised themselves remained elusive so far.
To address this question, the authors performed a very detailed analysis of the expression pattern of the components of the complex, both in wild-type and in mutant animals. Except for LIN-10, all others show dynamic expression during development. From the analysis of localisation of each of the members in mutants of the respective other genes the authors conclude that LIN-2 and LIN-7 depend on LIN-10 for their proper localisation on cytoplasmic punctae. LET-23 co-localises with LIN-7 on the basolateral plasma membrane and with the LIN-2/LIN-7/LIN-10 complex on intracellular punctae, which are likely to correspond to Golgi stacks or recycling endosomes. LET-23 can also be found on these punctae.

Given that the members of this complex and their role in the localisation of LET-23 have been published more than 20 years ago, it is surprising how little we still know about their specific in-vivo role during vulva development. Therefore, the data presented here shed more light on the how the complex may be built and how it may control the targeting of the receptor to the basolateral membrane. Given that the data are mostly obtained from localisation/expression of GFP-tagged proteins, the final conclusions are to some extend suggestions/speculations. But these can now be tested with more sophisticated methods.

Reviewer 3 Comments for the Author:
I have a few points that were not addressed:
a.The analysis is nearly exclusively based on fluorescently tagged proteins. Although the authors (or others) showed that these can rescue the respective mutant phenotypes, the localisation may still not reflect the “normal” pattern and dynamic, since immuno-fluorescent images only take snapshots.

We agree that a caveat to our studies are that tagged proteins might not show the same localization or dynamics as the endogenous untagged proteins. With regard to the lack of LIN-7a::EGFP localization to the basolateral membrane (Fig. S1) we do suggest in the results that the C-terminal tag could interfere with membrane localization. As suggested, we also added this caveat in the discussion with regards to a lack of LIN-2 or LIN-10 at the plasma membrane (Line 525). I interpret the reference to immune-fluorescent images as meaning that these could have been used as a complementary approach, which we agree could have strengthened the study, although they have their caveats as well.

b.In addition, some of these proteins are subjected to posttranslational modifications as shown in other systems, and the genes may encode more than one isoform.

We agree that PTMs and splice variants could be important factors regarding the formation and localization of the LIN-2/7/10 complex. For example, LIN-2b isoform that lacks the LIN-10 interaction domain did not interact with LIN-10, as expected, but does interact with LIN-7 (noted in Figure 7 and lines 324-327). We don’t know if LIN-2b isoform is expressed in the VPCs. If so, then some LIN-2 puncta that do not colocalize with LIN-10, might represent LIN-2B specific vesicles. This possibility was added to the discussion.

Regarding PTMs, LIN-10 has been shown to be phosphorylated and hydroxylated by Chris Rongo’s group. It would be interesting in future studies to specifically perturb these PTMs and see their effect on localization in the VPCs.

c.Furthermore, it has been shown previously that other molecules (ERM-1, EPS-8) are involved in some aspects of receptor trafficking/stabilisation. How does this relate to the LIN-2/LIN-7/LIN-10 complex?

© 2021. Published by The Company of Biologists under the terms of the Creative Commons Attribution License (https://creativecommons.org/licenses/by/4.0/).

Great point! We now discuss how EPS-8 interacts with LIN-2 and their potential role in endosome recycling/inhibition of internalization and how this might relate to our findings. ERM-1 is interesting in that it localizes to the basolateral membrane, like LIN-7, but interacts with a distinct pool of LET-23. Thank you for this point. In other organisms, both proteins are mutually exclusively associate with the Crumbs complex. This is something we now discuss as a possible point of functional interaction in the regulation of LET-23.

I suggest that the authors at least discuss these aspects.

Second decision letter

MS ID#: DEVELOP/2020/194167

MS TITLE: Golgi localization of the LIN-2/7/10 complex points to a role in basolateral secretion of LET-23 EGFR in the *C. elegans* vulval precursor cells

AUTHORS: Kimberley D Gauthier and Christian E Rocheleau

I have received all the reviewer comments, and as you will see the overall evaluation is positive and we would like to publish a revised manuscript in Development. However, reviewers 1 and 2 highlight some textual edits which will further improve the clarity of the manuscript. Please attend to all of the reviewers' comments in your revised manuscript and detail them in your point-by-point response. I do not expect to send the study back to the reviewers, it is important that you make the suggested edits. Please highlight all the textual changes in the revised manuscript. If you do not agree with any of their criticisms or suggestions explain clearly why this is so.

Reviewer 1

Advance summary and potential significance to field

LIN-2, -7, -10 are evolutionarily conserved, interacting proteins previously shown to promote basolateral localization of C. elegans LET-23/EGFR. In the 1998 Kaech et al publication providing evidence for this role, the question was left open as to whether LIN-2/7/10 influenced basolateral trafficking of LET-23 or acted at the basolateral plasma membrane to anchor LET-23 there or to prevent its endocytosis. In the 20+ years since then, the issue still has not been resolved. LIN-7 was reported to co-localize with LET-23 at basolateral membranes and junctions (based on overexpressed transgene reporters), but the location of the rest of the complex in vulva cells remained unclear. Here, Gauthier and Rocheleau revisit the question using endogenously expressed LET-23, LIN-2, LIN-7, and LIN-10 fusions (tagged using CRISPR genome editing). They visualize the localization of each fusion over developmental time, test for colocalization and for mis-localization in mutant backgrounds, and use the fusions for in vivo co-IP expts. The results clearly show that LIN-2, -7 and -10 are often found separately and co-localize in the Golgi and various endosomes rather than at basolateral membranes, and also reveal previously unknown LIN-2/LIN-7 subcomplexes and a LIN-10 --> LIN-2 --> LIN-7 regulatory hierarchy for Golgi recruitment. The results suggest a role for the complex in initial basolateral trafficking (or retrograde recycling) of LET-23 rather than in plasma membrane retention (though LIN-7 could play roles in both processes).

The Reviewers have responded satisfactorily to my prior comments, and to those of the other Reviewers. The addition of new data for Figure 9 allow more concrete conclusions about the site of LIN-2/LIN-7/LIN-10 overlap (Golgi), and changes to the text and Figure organization make the data and its impact and importance more clear.

© 2021. Published by The Company of Biologists under the terms of the Creative Commons Attribution License (https://creativecommons.org/licenses/by/4.0/).

Comments for the author

I have just a few minor comments about further clarifications:

1. Figures 1D,F,H, 2A, etc.: line labelled "BL" is really just basal, whereas the lateral membranes are not indicated, except by arrowhead in 1Dii. Perhaps Figures 1B, 2A could clarify. (Figure 2A is drawn as though nuclei are in a syncitium).

2. Figure 2I-2J: Please be more clear with graph labeling or legend, to specify that you are showing % of worms with this localization, not % of total fluorescence

Reviewer 2

Advance summary and potential significance to field

This revised manuscript from Gauthier and Rocheleau addresses many of the concerns raised by reviewers. Specifically, my concerns about clarity of narrative and the nature of the advances of this study are laid to rest, mostly through writing. Addition of Golgi markers strengthens a key observation. We now get a sense that while LIN-7 recognizes the C-terminus of LET-23, which we already knew, LIN-10 localizes to the Golgi and LIN-2 bridges the two. The observation comprises a significant advance in this field, which has not advanced substantially in the 20+ years since the characterization of the LIN-2/7/10 complex. In this context, the careful characterization of expression patterns and subcellular localization provides a strong foundation to understanding the functions of this complex and its context in broader development.

Comments for the author

Misc grammatical:

- in a couple of places “expression pattern” is conflated with “subcellular localization.” The former is intercellular, while the latter is subcellular. Ex lines 126, 127.
- “wildtype” should never be one word: “wild type” as a noun or “wild-type” as an adjective. , e.g. ln 138 would be “wild-type,” since it is adjectival
- “punctae” = plural, “puncta” = singular, e.g. ln 149 (most usages in this manuscript should be plural but are currently singular).
- Ln 500: “rescue vulval development?”

Reviewer 3

Advance summary and potential significance to field

Given that the LIN-2/LIN-7/LIN-10 complex and its importance for the localisation of LET-23 during development of the C. elegans vulva has been published more than 20 years ago, it is surprising how little we still know about their specific in-vivo role during vulva development. Therefore, the data presented here shed more light on the how the complex may be built and how it may control the targeting of the receptor to the basolateral membrane.

Comments for the author

The authors have carefully responeded to my suggestions raised in my previous report and have modified the text accordingly.

© 2021. Published by The Company of Biologists under the terms of the Creative Commons Attribution License (https://creativecommons.org/licenses/by/4.0/).

Second revision

Author response to reviewers' comments

Reviewer 1 Advance Summary and Potential Significance to Field:
LIN-2, -7, -10 are evolutionarily conserved, interacting proteins previously shown to promote basolateral localization of C. elegans LET-23/EGFR. In the 1998 Kaech et al publication providing evidence for this role, the question was left open as to whether LIN-2/7/10 influenced basolateral trafficking of LET-23 or acted at the basolateral plasma membrane to anchor LET-23 there or to prevent its endocytosis. In the 20+ years since then, the issue still has not been resolved. LIN-7 was reported to co-localize with LET-23 at basolateral membranes and junctions (based on overexpressed transgene reporters), but the location of the rest of the complex in vulva cells remained unclear. Here, Gauthier and Rocheleau revisit the question using endogenously expressed LET-23, LIN-2, LIN-7, and LIN-10 fusions (tagged using CRISPR genome editing). They visualize the localization of each fusion over developmental time, test for colocalization and for mis-localization in mutant backgrounds, and use the fusions for in vivo co-IP expts. The results clearly show that LIN-2, -7 and -10 are often found separately and co-localize in the Golgi and various endosomes rather than at basolateral membranes, and also reveal previously unknown LIN-2/LIN-7 subcomplexes and a LIN-10 --> LIN-2 --> LIN-7 regulatory hierarchy for Golgi recruitment. The results suggest a role for the complex in initial basolateral trafficking (or retrograde recycling) of LET-23 rather than in plasma membrane retention (though LIN-7 could play roles in both processes).

The Reviewers have responded satisfactorily to my prior comments, and to those of the other Reviewers. The addition of new data for Figure 9 allow more concrete conclusions about the site of LIN-2/LIN-7/LIN-10 overlap (Golgi), and changes to the text and Figure organization make the data and its impact and importance more clear.

Reviewer 1 Comments for the Author:
I have just a few minor comments about further clarifications:

1. Figures 1D,F,H, 2A, etc.: line labelled "BL" is really just basal, whereas the lateral membranes are not indicated, except by arrowhead in 1Dii. Perhaps Figures 1B, 2A could clarify. (Figure 2A is drawn as though nuclei are in a syncitium).

As suggested, we change BL in Figure 1 to B to be more specific. We added the lateral membranes to the diagram in Figure 2A and thus kept the BL label as this seemed appropriate.

2. Figure 2I-2J: Please be more clear with graph labeling or legend, to specify that you are showing % of worms with this localization, not % of total fluorescence

We revised the figure legend for Fig 2I and J to indicate that we are showing the % of worms imaged with punctate or membrane localization.

Reviewer 2 Advance Summary and Potential Significance to Field:
This revised manuscript from Gauthier and Rocheleau addresses many of the concerns raised by reviewers. Specifically, my concerns about clarity of narrative and the nature of the advances of this study are laid to rest, mostly through writing. Addition of Golgi markers strengthens a key observation. We now get a sense that while LIN-7 recognizes the C-terminus of LET-23, which we already knew, LIN-10 localizes to the Golgi and LIN-2 bridges the two. The observation comprises a significant advance in this field, which has not advanced substantially in the 20+ years since the characterization of the LIN-2/7/10 complex. In this context, the careful characterization of expression patterns and subcellular localization provides a strong foundation to understanding the functions of this complex and its context in broader development.

Reviewer 2 Comments for
the Author: Misc
grammatical:

•in a couple of places “expression pattern” is conflated with “subcellular localization.” The

© 2021. Published by The Company of Biologists under the terms of the Creative Commons Attribution License (https://creativecommons.org/licenses/by/4.0/).

former is intercellular, while the latter is subcellular. Ex lines 126, 127.

Thank you for identifying this. We improved our wording here and elsewhere in the paper (highlighted in yellow).

•“wildtype” should never be one word: “wild type” as a noun or “wild-type” as an adjective. , e.g. ln 138 would be “wild- type,” since it is adjectival

We were unaware of the noun versus adjective usage. We have changed this throughout.

•“punctae” = plural, “puncta” = singular, e.g. ln 149 (most usages in this manuscript should be plural but are currently singular).

There is conflicting information on the use of puncta, punctae and punctum online. We changed the usage as you suggest.

•Ln 500: “rescue vulval
development?” Corrected.

Reviewer 3 Advance Summary and Potential Significance to Field:
Given that the LIN-2/LIN-7/LIN-10 complex and its importance for the localisation of LET-23 during development of the C. elegans vulva has been published more than 20 years ago, it is surprising how little we still know about their specific in-vivo role during vulva development. Therefore, the data presented here shed more light on the how the complex may be built and how it may control the targeting of the receptor to the basolateral membrane.

Reviewer 3 Comments for the Author:
The authors have carefully responeded to my suggestions raised in my previous report and have modified the text accordingly.

Third decision letter

MS ID#: DEVELOP/2020/194167

MS TITLE: Golgi localization of the LIN-2/7/10 complex points to a role in basolateral secretion of LET-23 EGFR in the *C. elegans* vulval precursor cells

AUTHORS: Kimberley D Gauthier and Christian E Rocheleau
ARTICLE TYPE: Research Article

I am happy to tell you that your manuscript has been accepted for publication in Development, pending our standard ethics checks. With regards to your question on the usage of "puncta" vs "punctae" Development has used both variations in the past. For this manuscript, we will stick with 'punctae'. Thank you for addressing all the edits suggested by the reviewers.

© 2021. Published by The Company of Biologists under the terms of the Creative Commons Attribution License (https://creativecommons.org/licenses/by/4.0/).
